# Physical Activity, Exercise, and Rehabilitation in Myasthenia Gravis: A Systematic Review and Narrative Synthesis

**DOI:** 10.7759/cureus.114880

**Published:** 2026-08-20

**Authors:** Alicja Karbownik, Monika Lis, Aleksandra Hejnosz, Mateusz Szczerba, Hubert Piwar, Olga Grodzka, Magdalena Puziuk, Paweł Mikołajczak, Barbara Michałowicz, Patryk Cierpiał, Katarzyna Rode, Albert Staranowicz, Jan Pawłasek, Mateusz Sucharski, Martyna Dziurzyńska, Patryk Dziurzyński, Wiktor Niemczyk, Aleksandra Garbacik

**Affiliations:** 1 Department of Orthopaedics and Traumatology of the Musculoskeletal System, Medical University of Warsaw, Warsaw, POL; 2 Department of Medicine, Mazovian "Bródnowski" Hospital, Warsaw, POL; 3 Department of Medicine, Dr. Anna Gostynska Wolski Hospital, Warsaw, POL; 4 Doctoral School, Medical University of Warsaw, Warsaw, POL; 5 Department of Medicine, Dr. Aleksander Majkowski District Hospital, Kartuzy, POL; 6 Department of Medicine, National Medical Institute of the Ministry of the Interior and Administration, Warsaw, POL; 7 Department of Medicine, Wladyslaw Bieganski District Hospital in Ilawa, Iława, POL; 8 Department of Medicine, Central Clinical Hospital in Warsaw, Warsaw, POL; 9 Department of Pharmacotherapy and Pharmaceutical Care, Medical University of Warsaw, Warsaw, POL; 10 Department of Medicine, Military Institute of Medicine - National Research Institute, Warsaw, POL; 11 Department of Internal Medicine and Gastroenterology, Mazovian "Bródnowski" Hospital, Warsaw, POL; 12 Department of Gastroenterology, Bielanski Hospital, Warsaw, POL

**Keywords:** breathing exercises, exercise therapy, fatigue, myasthenia gravis, quality of life, rehabilitation

## Abstract

Myasthenia gravis causes fluctuating, fatigable weakness, creating uncertainty about physical activity and exercise. This systematic review examined habitual physical activity and sedentary behaviour, barriers and facilitators to exercise, structured exercise, respiratory muscle training, and physical rehabilitation in autoimmune myasthenia gravis. PubMed, Scopus, and Web of Science Core Collection were searched from inception to 30 July 2026, without database date or language filters; backward citation searching was completed on 1 August 2026. Primary human studies published in English were eligible if they reported a relevant measure of physical activity, a defined physical intervention, or barriers, facilitators, or professional advice as an explicit primary focus. We included randomized trials, non-randomized intervention studies, observational and qualitative studies, and case series of at least two participants. Multiple reports from the same cohort or trial were linked before synthesis. Risk of bias was assessed with tools appropriate to each design, and certainty was rated by outcome domain. Of 2,767 database records imported into EndNote (Clarivate, Philadelphia, PA), 1,946 remained after deduplication. Across database and citation searching, 66 reports were assessed in full, and 47 reports representing 38 study units (whole studies or distinct study components) were included. In several observational datasets, greater activity was associated with less fatigue or lower disease burden; however, most analyses were cross-sectional and vulnerable to confounding and reverse causality. Intervention studies were small and heterogeneous. Some secondary outcomes and results measured after treatment favoured the intervention for respiratory muscle performance, functional capacity, strength, or patient-reported outcomes, and some measures improved within groups; however, several controlled primary outcomes were null. Evidence from perioperative care, recovery after crisis or hospitalization, and paediatric populations addressed distinct clinical contexts and was not generalized to stable community-dwelling adults. Some selected cohorts achieved high completion or attendance, but reporting of adverse events was incomplete, and certainty about safety was very low. Individually adapted activity and exercise appeared feasible for many selected participants with clinically stable, predominantly mild-to-moderate disease. However, efficacy remains very uncertain, and safety has not been established across the broader myasthenia gravis population.

## Introduction and background

Myasthenia gravis (MG) is an autoimmune disease of the neuromuscular junction. Antibodies most often target the acetylcholine receptor and, in smaller subgroups, muscle-specific kinase or low-density lipoprotein receptor-related protein 4. Impaired neuromuscular transmission causes fluctuating weakness of ocular, bulbar, respiratory, axial, or limb muscles; weakness may remain localized or become generalized [1].

Treatment is tailored to the MG subgroup and may include acetylcholinesterase inhibitors, immunotherapy, and thymectomy. Intravenous immunoglobulin or plasma exchange may provide rapid treatment during exacerbations, and respiratory failure may require intensive care [1,2]. Nationwide registry data from Denmark, Finland, and Sweden showed a substantial increase in annual MG prevalence per 100,000 people between 2000 and 2020 [3]. This epidemiological increase provides context for examining long-term function and participation alongside acute disease control.

For this review, we distinguished four related but analytically separate constructs. Physical activity referred to bodily movement that increases energy expenditure. Exercise referred to planned, structured, and repetitive activity intended to improve or maintain fitness. Respiratory muscle training referred to a specific load or endurance stimulus applied to inspiratory muscles, expiratory muscles, or both. Rehabilitation referred to a broader multimodal programme that could combine exercise with respiratory care, education, energy conservation, psychological support, or perioperative recovery strategies.

These operational distinctions matter in MG. A cross-sectional association between habitual activity and fatigue does not estimate the effect of an exercise prescription, and perioperative pulmonary rehabilitation does not answer the same question as long-term training in stable outpatients.

Historically, clinicians were reluctant to encourage exercise in MG because overwork was thought to aggravate weakness. This “overuse weakness” hypothesis has not been confirmed in controlled studies [4]. Sedentary behaviour and physical deconditioning may in turn contribute to reduced fitness and fatigue [4]. Earlier reviews challenged blanket avoidance of exercise but emphasized that the evidence came from small, heterogeneous studies conducted mainly in selected patients with mild-to-moderate or stable disease [4,5].

A 2024 scoping review identified 24 studies, including interventional and observational designs, spanning aerobic exercise, resistance exercise, balance training, and stretching; adverse events related to exercise were reported in two studies [6]. Subsequent reviews addressed narrower evidence sets. Xiangxiang et al. included six studies of physical or respiratory rehabilitation [7]. Gutierrez-Del-Val et al. included 20 studies of physical activity or training, 10 of which contributed to quantitative meta-analyses [8]. Vinciguerra et al. included eight controlled intervention studies and used narrative synthesis because clinical and methodological heterogeneity precluded meta-analysis [9]. The present review updates the search through 30 July 2026 and extends the scope to habitual physical activity, digital activity monitoring, barriers and facilitators, and rehabilitation in distinct clinical settings. Across this literature, overlapping publications from the same trial or cohort require linkage, changes observed within groups must be distinguished from comparisons between groups, and cross-sectional associations cannot establish causality.

This review addressed two linked but analytically separate questions. The first concerned habitual physical activity and sedentary behaviour: How are they distributed among people with MG, how are they associated with fatigue, quality of life, disease severity, and physical function, and what barriers, facilitators, and professional advice are reported? The second concerned structured physical interventions: What effects, adherence, tolerability, and adverse events are reported in MG?

Intervention evidence was stratified into stable community-dwelling adults, respiratory muscle training, perioperative or post-acute rehabilitation, and juvenile MG. The review also reconciled overlapping reports, verified numerical extractions against full texts, recorded disease stability and treatment information, and assessed risk of bias and certainty by evidence domain.

## Review

Materials and methods

Reporting Guidance and Registration

This systematic review was reported in accordance with the Preferred Reporting Items for Systematic Reviews and Meta-Analyses (PRISMA) 2020 statement. The literature search was additionally guided by the PRISMA extension for reporting literature searches (PRISMA-S) [10,11]. Applicable reporting items are addressed in the manuscript and Appendices. The review was not prospectively registered, and no prospective review protocol was prepared.

Accordingly, the eligibility criteria, search methods, report linkage, risk of bias assessment, and certainty framework were not constrained by a prespecified protocol and should be regarded as post hoc. The methods used in the review are described below.

Review Questions and Eligibility Criteria

The first review question used a population, exposure, comparator, and outcome framework. In people with autoimmune MG, how do measured habitual physical activity or sedentary behaviour relate to fatigue, MG severity, quality of life, functional capacity, or participation when compared with lower activity, a group without MG, or another time point in the same person? It also asked what barriers, facilitators, or professional advice were reported when these were an explicit primary study focus.

The second review question used a population, intervention, comparator, and outcome framework. In people with autoimmune MG, what effects and harms are reported after a defined exercise, respiratory muscle training, or rehabilitation intervention compared with usual care, rest, another physical programme, no intervention, or baseline?

We included English-language, peer-reviewed reports of primary human studies involving people with autoimmune MG, including juvenile autoimmune MG. Randomized and non-randomized intervention studies, prospective or retrospective cohorts, analytical or descriptive cross-sectional studies, qualitative studies, and case series with at least two participants were eligible.

Reports had to quantify habitual physical activity or sedentary behaviour, evaluate a defined physical intervention, or focus explicitly on barriers or facilitators to physical activity or exercise or on professional advice. At least one MG-relevant clinical, functional, respiratory, patient-reported, adherence, or safety outcome, or an outcome concerning barriers or facilitators, had to be reported. Items concerning exercise that were incidental to a broader survey of disease burden, health beliefs, or general rehabilitation needs did not by themselves establish eligibility. There was no publication date restriction.

We excluded congenital myasthenic syndromes, animal studies, and single-patient reports. Reviews, editorials, protocols, and conference abstracts without a citable full report were also excluded, as were pharmacological or surgical studies without a defined physical component.

We additionally excluded studies that only validated an exercise test or outcome measure and did not include a habitual activity exposure or training intervention. Studies of mixed diagnoses were excluded when data for participants with MG could not be extracted separately. Eligibility required a full report in English. Records known before retrieval to lack such a report were excluded during title and abstract screening; retrieved reports in languages other than English were excluded when assessed in full. Reviews were used only for background and citation checking.

Information Sources and Search Strategy

PubMed, Scopus, and Web of Science Core Collection were searched from database inception to 30 July 2026. Searches combined controlled vocabulary, where available, and title/abstract/topic terms for MG with terms for exercise, physical activity, sedentary behaviour, aerobic and resistance training, respiratory muscle training, breathing exercise, physiotherapy, rehabilitation, walking, treadmill, cycling, balance, stretching, sport, and activity monitoring. No date, language, human, document type, or study design filter was applied in the database interfaces. Trial registries were not searched as an independent route for identifying additional studies, and no separate grey literature search was performed.

However, registry records for included studies were checked when an identifier was reported or could be matched. These records were used to compare dates, prespecified outcomes, and intervention details [12-15]. PubMed returned 768 records, Scopus 1,169 records, and Web of Science 830 records. Backward citation searching of included reports and the six prior reviews cited in the Introduction [4-9] was completed on 1 August 2026.

Results from all three databases were exported in their native bibliographic formats and imported into a single EndNote library (Clarivate, Philadelphia, PA). EndNote was used for record management and deduplication. Potential duplicates were reviewed by comparing titles, authors, publication years, digital object identifiers (DOIs), and PubMed identifiers (PMIDs). Reference lists of included articles and those six prior reviews were then checked to identify related publications and potentially missed primary reports. This process identified one additional eligible report, Anggarini et al. [16], in the reference list of the included Amalina report [17]. It was recorded in the pathway for studies identified through other methods in the PRISMA accounting. Search and deduplication counts were retained in the review records.

Selection Process and Management of Overlapping Reports

After deduplication in EndNote, titles and abstracts were screened against the eligibility criteria. Potentially relevant reports were then assessed in full. Eligibility decisions and reasons for exclusion after full assessment were recorded. Publications that might have described the same study were retained for assessment of possible report linkage rather than removed as duplicates.

Reports were linked when full texts showed the same registry or ethics identifier, recruitment site and period, sample, intervention, and baseline characteristics. Related publications were counted as separate reports but as one study. A new intervention episode was treated as a distinct study, even when some participants had taken part in an earlier programme. A report containing two distinct samples was mapped to two study components. A pooled secondary analysis of existing cohorts did not create a new study.

Data Collection Process and Data Items

A structured extraction form was applied to all included reports. We extracted the citation, country and setting, study design, recruitment period, sample size and attrition, MG definition and subtype, Myasthenia Gravis Foundation of America (MGFA) class or another severity measure, and explicit criteria for disease stability. We separately recorded the intervention or exposure, comparator, duration, follow-up, medication regimen and changes, and assessment timing relative to pyridostigmine when reported.

We also extracted prespecified outcomes, adjusted and unadjusted estimates, adherence, withdrawals, protocol modifications, symptom worsening, falls, respiratory deterioration, MG exacerbation or crisis, hospitalization, serious and non-serious adverse events, funding, and conflicts of interest. Sponsorship and investigator conflicts were considered qualitatively when interpreting reporting bias; neither funding nor a disclosed conflict was treated as proof of bias on its own.

For controlled studies, estimates comparing groups were prioritized over changes observed within groups. For observational studies, adjusted estimates were prioritized, with the adjustment set recorded. For uncontrolled studies, change within participants was retained but identified explicitly as such. When reports conflicted, values were checked across the complete report and trial records and, where possible, by recalculation. Values that could not be reconciled were flagged and not used as precise estimates of effect. The latest prespecified end-of-intervention assessment was used for the principal synthesis; later follow-up was described separately.

Outcomes and Effect Measures

The principal outcome domains were MG severity or Myasthenia Gravis Activities of Daily Living (MG-ADL), fatigue and MG-specific quality of life, respiratory muscle performance and pulmonary function, objective physical function or exercise capacity, and safety. Additional outcomes included habitual activity or sedentary dose, muscle strength, psychological outcomes, participation, adherence, withdrawal, treatment change, and barriers, facilitators, or professional advice.

For continuous outcomes, we preferred mean differences between groups or differences in change with confidence intervals. Standardized effects were considered only when instruments differed, and estimates were clinically comparable. For binary outcomes, we sought risk ratios or event proportions. For studies of exposure and outcome, we extracted adjusted regression coefficients, odds ratios, or correlations. A change within a group before and after intervention was never labelled a treatment effect or a mean difference between groups.

Risk of Bias

Risk of bias was assessed for the principal numerical results selected for synthesis rather than assigned once to an entire publication. When patient-reported and objectively measured results, outcome completeness, or eligible analyses differed materially within one study, separate rows were used for the relevant results.

Results from individually randomized parallel-group trials were assessed for the effect of assignment using the 22 August 2019 version of the Cochrane Risk of Bias 2 (RoB 2) tool [18]. The permitted overall judgments were low risk, some concerns, or high risk. Results from the two-sequence, two-period crossover trial and the acute within-participant comparison with randomized test order were assessed using the 18 March 2021 RoB 2 crossover variant, including domain S for period and carryover effects. For the acute comparison, the assessment concerned the randomized order of rhythmic auditory stimulation and no stimulation, not the comparison between cohorts tested at 100% and 110% of cadence.

The randomized trial of contralateral limbs was not treated as a crossover trial. As no dedicated RoB 2 framework is available for interventions assigned to body regions, we used an explicitly adapted, unvalidated version. Additional considerations included paired analysis, the unit of analysis, cross-education or spillover, and contamination [18,19].

Non-randomized reports that appeared to provide a comparative estimate of an intervention effect were assessed using the original 2016 Risk Of Bias In Non-randomized Studies of Interventions (ROBINS-I) tool. For each result, we specified the target comparison and the estimand supported by the full report. An effect of assignment at baseline was retained only when intervention strategies could be defined at a common time zero. Accordingly, Ambrogi was framed as an as-treated comparison of selected matched completers with historical controls [20].

The study by Freitag et al. was evaluated as an observed comparison restricted to complete cases [21]. The intended comparative change effect in Hsu et al. could not be estimated reliably because only separate tests within each group were presented [22]. For Otaka et al., rehabilitation intensity accumulated after ventilator weaning and could depend on recovery; the assessment therefore concerned an observational association rather than an effect of assignment at baseline [23]. Potential confounders relevant to each context included baseline MG severity and phenotype, functional and activity status, MG treatment, comorbidities, motivation, clinical indication, and recovery trajectory. Ordinary adherence or clinical tailoring was not automatically treated as a deviation from the intended intervention, whereas an exposure that depended on recovery could create critical confounding or misclassification of the intervention [24].

Uncontrolled changes measured before and after an intervention were assessed with the 2024 Joanna Briggs Institute (JBI) checklist for quasi-experimental studies [25]. For studies by Punga et al. and Wong et al., the case series appraisal was retained for feasibility, tolerability, and adverse events, whereas efficacy results measured before and after intervention received a separate quasi-experimental appraisal [26,27]. Linked secondary reports were appraised according to the design of the result used. Thus, the RESTOREX subanalysis limited to the intervention group [28,29], the MGEX accelerometry report based on data collected before randomization [30], and the pooled O'Connor baseline report [31] did not inherit the judgment of the parent intervention study.

Analytical cross-sectional results were assessed using the 2026 JBI analytical cross-sectional tool and cohort results using the 2025 JBI cohort tool; case series and the linked qualitative companion report were assessed with the relevant JBI 10-item checklists [32-34]. JBI responses were not summed. The overall concern labels used in this review describe the importance of limitations at the item level and are not official JBI scores or categories. Purely descriptive results were labelled as such and were not given a causal interpretation.

The appraisal register contained 75 assessments: 34 RoB 2, four ROBINS-I, 17 JBI cross-sectional, five JBI cohort, three JBI case series, one JBI qualitative, and 11 JBI quasi-experimental assessments. Separate rows for different results do not represent additional studies and do not change the count of 38 study units. Complete worksheets for the signalling questions were retained in the review records.

Synthesis and Certainty Assessment

Clinical settings, interventions, designs, outcomes, instruments, follow-up periods, and analysis methods were too heterogeneous to support a defensible pooled estimate. Several publications also arose from overlapping trials or cohorts. We therefore performed a structured narrative synthesis, grouped by review question and clinical context. We emphasized the direction and magnitude of controlled effects; uncontrolled change, cross-sectional association, and qualitative findings were presented separately. The qualitative companion report was synthesized descriptively by recurring barrier, facilitator, and advice categories rather than being used to estimate an intervention effect. Null and harmful findings were retained, and conflicting numerical data were not resolved by choosing the more favourable value.

Certainty was assessed for each prespecified principal outcome within each clinical context using the traditional Grading of Recommendations Assessment, Development, and Evaluation (GRADE) approach. GRADE was applied to bodies of evidence rather than individual studies and remained applicable without a pooled estimate [35-38]. The target of each rating was confidence in the direction and clinically important magnitude of the narrative effect statement. Evidence from randomized trials and from non-randomized intervention studies was initially assessed in separate streams. Randomized evidence began at high certainty, whereas evidence from non-randomized interventions and studies of associations between exposure and outcome began at low certainty.

Non-randomized intervention evidence was used only as complementary contextual evidence when it addressed the same population, intervention, comparator, and outcome. As the randomized and non-randomized estimates were too heterogeneous to assess agreement reliably, they were not combined, and the non-randomized evidence did not automatically increase or reduce the certainty rating for randomized evidence. RoB 2 and ROBINS-I assessments for the results contributing to each body informed the GRADE risk of bias domain. Non-randomized evidence began at low certainty; confounding and selection inherent in the absence of randomization were not counted again as an additional downgrade, although other serious study limitations could support further downgrading.

We considered risk of bias, inconsistency, indirectness, imprecision, and publication or dissemination bias, together with the potential upgrading factors of a credible large effect, dose-response gradient, or residual confounding expected to attenuate rather than explain an observed association. No body met an upgrading criterion. Inconsistency was assessed only across sufficiently comparable outcomes and clinical contexts and was not downgraded when a body contained only one study. When available, confidence intervals were judged against the null and clinically important effects. Otherwise, imprecision was judged from participant or event counts and the range of effects that remained plausible.

A body of evidence was not downgraded solely because it contained one study. No body of evidence for an outcome contained enough clinically and methodologically comparable independent estimates for meta-analysis, funnel plots, or formal asymmetry tests. Possible bias due to missing evidence was assessed qualitatively by comparing registry entries, protocols, and reports; considering known missing outcome results; and looking for patterns of small positive studies when missing null or unfavourable evidence was plausible. Selection among reported measurements or analyses was handled in the risk of bias assessment for each study and was not counted again as publication bias; small sample size was handled under imprecision. The single unretrieved report from a mixed population with neuromuscular disease was retained as a search limitation but was not treated as evidence of publication bias because its eligibility and the availability of separate MG results were unknown.

Results

Study Selection

The database searches yielded 2,767 records: 768 from PubMed, 1,169 from Scopus, and 830 from Web of Science. All records were imported into EndNote. After 821 duplicate records were removed in EndNote, 1,946 unique records underwent title and abstract screening. Sixty-six database reports were sought for retrieval. One 1993 report on ventilatory training in a mixed population with neuromuscular disease by Gross et al. could not be retrieved despite searches of publisher records and library holdings and attempts to contact the authors.

**Figure 1:**
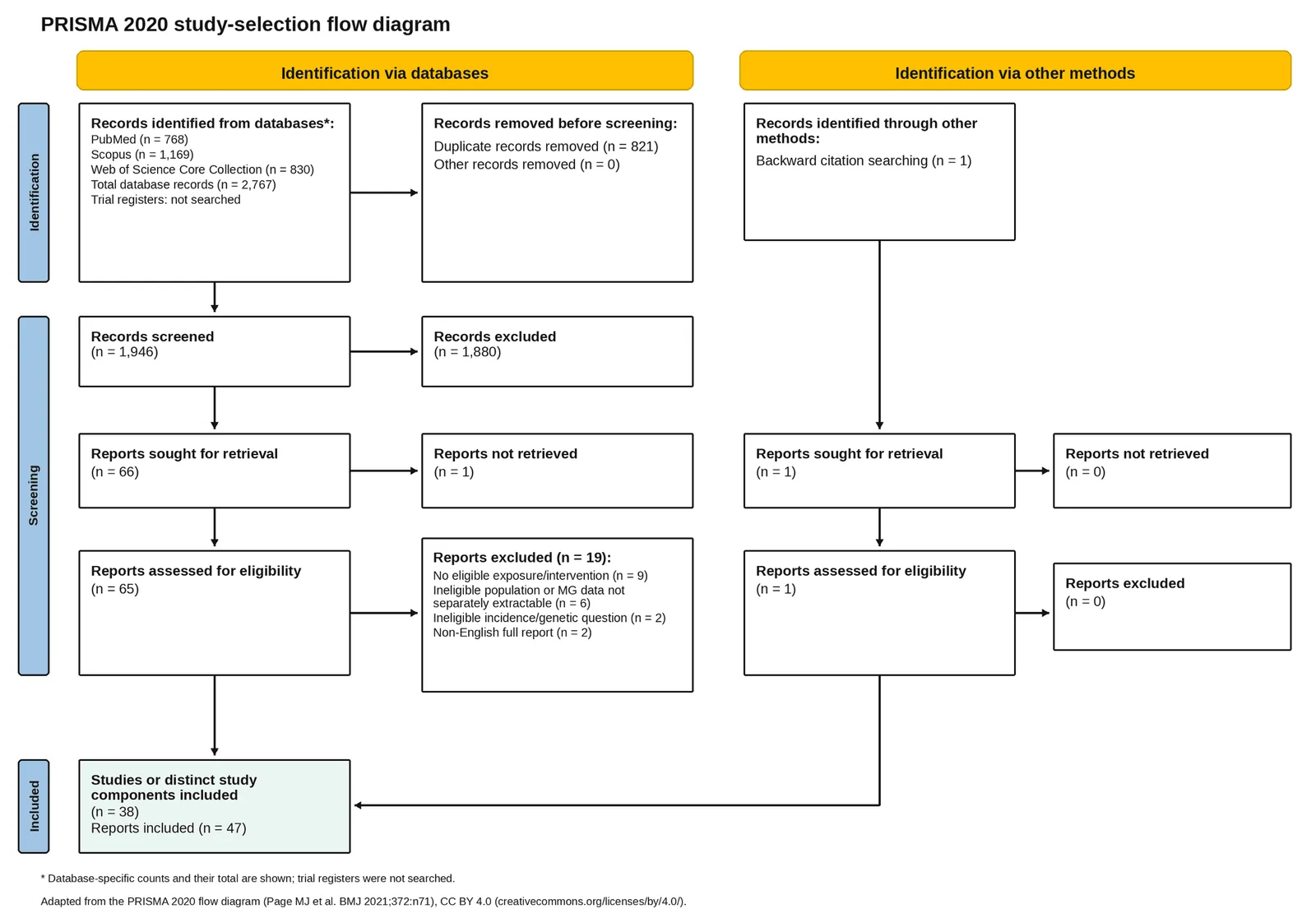
PRISMA 2020 flow diagram of study selection. Database searches yielded 2,767 records, and backward citation searching identified one additional report. Overall, 47 reports representing 38 study units were included.

Sixty-five reports identified through database searches were assessed, and 19 were excluded. Nine lacked an eligible exposure or intervention; six had an ineligible population or no separately extractable MG data; two addressed an ineligible incidence or genetic question; and two were full reports in languages other than English. The database pathway therefore contributed 46 included reports. Backward citation searching identified one additional eligible report, which was retrieved and assessed without exclusion. Across both pathways, 67 reports were sought, 66 were assessed, and 47 reports representing 38 study units were included in the narrative synthesis. Figure 1 presents the PRISMA flow diagram.

Study and Report Characteristics

The 47 included reports, published between 1993 and 2026, covered randomized trials, controlled and uncontrolled non-randomized intervention studies, longitudinal and cross-sectional observational studies, retrospective administrative cohorts, a linked qualitative companion report on barriers and advice, and small case series. Sample sizes ranged from two participants in a perioperative case series to several hundred participants in national surveys and administrative cohorts. Most training studies in community settings enrolled selected adults with stable, mild-to-moderate generalized MG. Evidence for ocular MG, severe or unstable disease, major bulbar or respiratory compromise, and substantial cardiopulmonary comorbidity was sparse.

Linking related reports reduced the 47 included reports to 38 study units. Reports were grouped using explicit trial links, ethics identifiers, recruitment sites and dates, characteristics of individual participants or groups, intervention methods, and exact source statements. Confirmed links were distinguished from possible overlap. Denominators from each report were retained, and uncertain relationships were not classified as either wholly independent or fully overlapping.

Six publications from the Surabaya Centre were assigned to three trial families. Four reports shared ethics approval (0601/KEPK/II/2023), the same setting, closely similar intervention and comparator programmes, and substantially overlapping dates; they were treated as one apparent parent trial, although their randomized and analysed denominators were irreconcilable [17,39-41].

Kartika et al. and Anggarini et al. had different ethics approvals and nonidentical flows, so they were not formally merged [16,42]. However, their final intervention groups each contained nine participants with the same reported mean age and standard deviation (47.78 ± 5.36 years) and the same sex distribution (two men and seven women) [16,42]. Partial or substantial participant overlap is therefore probable but could not be confirmed. The synthesis treats them as separate trial components with an explicit non-independence warning.

Two other groups of publications were explicitly linked. The Benefits and Tolerance of Exercise in Myasthenia Gravis (MGEX) trial produced an accelerometry analysis conducted before randomization and a randomized home rowing report [30,43]. The Rest or 30-Min Walk as Exercise Intervention (RESTOREX) trial produced a primary randomized report and a linked biomarker and clinical subanalysis of 15 participants [28,29]. The 12 healthy controls in the latter provided only baseline biomarker comparisons and were not treated as a second comparative MG study [28,29].

Participant overlap also affected publications on respiratory training and reports from Sweden, Denmark, and Australia. Nine of the 10 participants in Rassler et al. (2011) could be matched directly to participants labelled TG1-TG9 in the later Freitag cohort. Five had already participated in the 2007 episode, and the final Freitag cohort contained seven previous participants, including one who later entered as a control [21,44,45].

O'Connor et al. pooled the two complete baseline samples from the Uppsala (n = 14) and Jönköping (n = 13) intervention cohorts; the pooled sample was therefore not counted as a third cohort [31,46,47]. The Swedish survey reports reused the same 40 Jönköping respondents, and possible overlap between that survey sample and the 13-person Jönköping exercise cohort could not be reconstructed [46,48,49]. Punga et al. recruited mainly from the earlier Uppsala cohort, but the exact number who had participated previously was not reported [26,47].

Andersen and Vissing reanalysed the Danish national survey and additionally described a 69-patient clinic component; participant overlap between these components was not reported [50,51]. The Alsop qualitative report selected 10 participants from the earlier 85-person survey project [52,53], and Thomsen et al. followed participants from two earlier Vinge baseline cohorts rather than creating a new baseline population [54].

Risk of Bias Across the Evidence Base

Risk of bias was a dominant limitation across all evidence streams. Judgments for randomized studies differed by result: patient-reported outcomes from participants who knew their allocation, outcomes with substantial missing data, and results affected by selective reporting were commonly at high risk, whereas some objective results had some concerns. No certainty rating for randomized evidence was upgraded on the basis of favourable uncontrolled or observational findings. Among the four ROBINS-I assessments, Otaka, Hsu, and the Leipzig RMET result after long-term follow-up were at critical overall risk of bias; Ambrogi's comparison of length of stay among selected matched completers remained at serious risk.

The principal concerns were confounding and reverse causality, selection of analysed participants, definition of exposure after baseline, attrition related to prognosis, measurements influenced by participant effort, invalid or absent comparative analyses, and selective reporting. Every efficacy assessment from an uncontrolled single-group study was judged to have a high concern because neither a concurrent control nor an adequate repeated baseline separated the intervention from natural history, regression to the mean, additional care, or time effects. Most analyses of habitual activity were cross-sectional or otherwise vulnerable to self-reported exposure, attrition, limited adjustment, uncertain causal direction, selective availability of device data, dependence among observations, and internally inconsistent source data. Linked secondary analyses were appraised according to their actual design.

Habitual Physical Activity, Sedentary Behaviour, and Barriers

Studies using accelerometers or questionnaires reported heterogeneous activity patterns in selected MG samples. These included substantial variation between participants in activity measured by devices and differences in whether physical activity and sedentary behaviour thresholds were met [30,31,52,55,56]. Adjusted inverse associations between self-reported activity and fatigue were reported in Danish national and Dutch registry surveys [50,57]. Earlier reports from Sweden and Estonia compared participants with MG with matched controls or compared national cohorts. They reported differences in activity patterns but did not estimate an adjusted association within the MG population between activity and fatigue or another patient-reported outcome [48,49]. Grohar-Murray et al. reported no significant overall relationship between fatigue scores and self-care actions. Among 36 respondents who used low-impact aerobic exercise, 25 considered it helpful, and 11 did not; fatigue scores did not differ significantly by perceived helpfulness [58].

Cross-sectional studies cannot establish direction. People with less fatigue, lower MG severity, fewer comorbidities, better mobility, or more favourable social circumstances may be more able to exercise. In the 12-week Dimmick cohort, 30 participants were enrolled, but only 16 had sufficient data for the final analyses. Models centred on the cohort mean did not isolate change within each participant. They associated fewer steps, less moderate-to-vigorous and light activity, and more inactivity with worse fatigue or MG symptom ratings on the same day. In models scaled within each participant, the only retained daily behavioural association was between more moderate-to-vigorous activity and less fatigue; no daily activity or sleep variable was retained for symptom ratings. Individual estimates varied in direction, and one reported dispersion value and two reported confidence intervals were internally implausible [55].

A decentralized smartphone study related optional platform-derived step counts to MG-ADL scores and self-reported exacerbation status recorded on the same day. Only 26 participants contributed data to the analysis relating step count to MG-ADL, and 14 to the analysis relating exacerbation status to step count. These analyses did not account for repeated observations within participants; harmonization across devices or platforms, criteria for wear time, and thresholds for completeness of step data were not reported. The ClinicalTrials.gov record listed audiovisual symptom recording as a primary outcome and MG-specific quality of life as a secondary outcome; neither was reported in the article [12,56]. Over a mean follow-up of 4.6 years, Thomsen et al. found no change in the self-reported Physical Activity Scale score among participants with MG (p = 0.393) [54]. Physical Activity Scale scores did not differ significantly between the MG and control groups at either visit.

Unadjusted Pearson analyses among completers found a weak correlation between changes in activity and shoulder strength (r = 0.324, p = 0.01). Changes in activity did not correlate with changes in knee strength, 400-m walk performance, or the 30-second sit-to-stand test; the correlation with change in ankle strength was not reported [54].

The Alsop survey adjusted only for age, body mass index, and disease duration. Meeting its self-reported threshold of at least 500 metabolic equivalent minutes per week was associated with lower fatigue, better MG-specific quality of life, less walking limitation, and greater balance confidence. Sedentary time reported as less than 10 versus at least 10 hours per day was not associated with any of these outcomes. In the joint analysis, participants with insufficient activity and high sedentary time had greater fatigue, poorer MG-specific quality of life, more walking limitation, and lower balance confidence than the reference group with sufficient activity and less sedentary time. Participants with insufficient activity and less sedentary time had greater fatigue, poorer MG-specific quality of life, and more walking limitation than the reference group, but balance confidence did not differ. Several reported estimates contained incompatible coefficients, confidence intervals, or p-values, so those exact estimates were not interpreted [52].

In the Danish registry survey, each one-category increase in the modified Saltin-Grimby Physical Activity Level Scale was associated, after multivariable adjustment of complete cases, with lower scores in all five Multidimensional Fatigue Inventory domains. In the sensitivity analysis restricted to participants who were treated and regularly followed by a neurologist, the estimate for mental fatigue was null while the other four associations remained. Andersen and Vissing subsequently reanalysed 691 complete cases from the same survey of 779 respondents using the International Physical Activity Questionnaire and also reported a separate clinic accelerometer sample of 69 patients [50,51]. In the cross-sectional registry survey by Ruiter et al., one or more sessions of strenuous activity per week were associated with lower fatigue scores than no strenuous activity in the adjusted model of 397 participants. No category of daily moderate activity was associated with fatigue. The model included demographic and clinical variables, depressive symptoms, sleep quality, autoimmune comorbidity, and both activity measures, but not MG treatment [57].

Glaubitz et al. reported unadjusted inverse Spearman correlations of a study-specific metabolic-equivalent score with the Quantitative Myasthenia Gravis (QMG) score (r = -0.255, p = 0.019) and MG-ADL (r = -0.316, p = 0.004). No validation of the questionnaire was reported, and no treatment adjustment was made despite frequent corticosteroid, other immunosuppressive, and intravenous immunoglobulin use. Some quantities in the Glaubitz et al. report were labelled as steps per day. However, the questionnaire asked for self-reported daily walking distance in metres, optionally informed by a tracker, and did not report a conversion from metres to steps [59].

In qualitative interviews with 10 Australian adults with generalized MG, participants described fatigue, muscle weakness related to activity, and pain as barriers to physical activity, although the relation of pain to MG itself was uncertain. Effective medical management, social support, pacing and rest strategies, and perceived benefits to general health or MG facilitated activity, whereas professional advice was often considered inadequate or poorly tailored to MG [53].

In a French online survey, perceived barriers included health or symptoms, lack of supervision by an MG specialist, fear of adverse disease or symptom consequences, weather, insufficient exercise knowledge, low motivation, time, company, and practical access. Reported facilitators included expected or experienced health and symptom benefits, specialist supervision or coaching, exercise information, practical access or affordability, company, and support from family or healthcare professionals [60]. Birnbaum et al. classified 244 of 455 participants (53.6%) as exercisers and 211 (46.4%) as non-exercisers; the abstract reported 54% and 47%, whereas one sentence in the Results reversed the group proportions. The internally consistent counts of 244 and 211 were therefore retained. As these data were self-reported and cross-sectional, and the survey comparisons were univariate, they describe perceived determinants and associations rather than effects of prescribing exercise.

Structured Exercise in Selected Community-Dwelling or Outpatient Adults

Controlled trials produced mixed findings across heterogeneous primary outcomes. DIG-MG randomized 81 participants; prespecified omnibus tests were null for MG-ADL and the Fatigue Severity Scale, and a nominal pairwise MG-ADL difference favoured passive monitoring rather than physical activity coaching [61]. MGEX randomized 43 participants; home rowing did not improve the prespecified primary MG-specific quality of life outcome at month six, and nominal findings for MG-ADL and six-minute walk distance at month six were not sustained at month nine [43]. RESTOREX randomized 40 participants and analysed 38 completers.

The published MGQOL15 responder result, defined as improvement of more than 50%, favoured walking over rest. However, registration followed the trial; the sample size calculation addressed continuous change, multiple outcomes, time points, and responder thresholds were available, and the linked 15-person report could not be reconciled with the parent responder counts [13,28,29]. The linked RESTOREX biomarker report retained only a selected uncontrolled walking group subset; healthy controls supplied baseline biology rather than a controlled exercise effect. Rahbek et al. compared resistance with aerobic training rather than exercise with usual care and analysed only 12 of 15 randomized participants [62]. These results do not establish a consistent patient-centred benefit of structured exercise.

The Surabaya programme reported walking, fatigue, MG-ADL, disease severity, depression, pulmonary outcomes, and outcomes measured by surface electromyography across six publications [16,17,39-42]. Four later reports were linked as one apparent ethics-0601 trial family, while Kartika and Anggarini were retained as separate components with probable but unconfirmed participant overlap. Positive findings frequently arose from analyses limited to completers, tests of change within groups, or endpoints measured after treatment rather than clearly reported comparisons of randomized groups.

Kartika's Fatigue Severity Scale data were inconsistent. Anggarini excluded five of 23 randomized participants and reported ambiguous pooled deltas. Amalina emphasized favourable endpoints despite stating that changes did not differ significantly between groups. Deliana et al. did not adequately describe normalization or processing of surface electromyography or assessor blinding [39]. Small samples, attrition, unblinded assessment, effort-dependent or patient-reported outcomes, multiple publications presenting separate outcomes, unresolved cohort independence, and irreconcilable denominators produced high risk of bias for all principal Surabaya efficacy results.

Small uncontrolled programmes and case series reported selected improvements within participants. Without concurrent controls, however, these findings could not distinguish an intervention effect from natural fluctuation, regression to the mean, learning from repeated testing, additional care, or volunteer selection [26,27,46,47,63,64]. In Chang et al., 35 participants entered and 34 completed. Participants chose their weekly exercise dose, and two groups of 17 were formed after the analysis at the median of 56.3 minutes per week; this was not a randomized comparison of exercise dose [63]. Farrugia et al. enrolled 10 participants in a combined physical and psychological fatigue management programme and analysed nine after baseline. Only the Visual Analogue Fatigue Scale improved during treatment, and the improvement was not sustained [64]. Punga et al. analysed seven completers recruited mainly from the earlier Westerberg Uppsala study, although the exact number reused was not reported [26,47].

The Westerberg Uppsala cohort (14 entered and 11 completed) and Jönköping cohort (13 entered and 10 completed) were separate; O'Connor et al. later pooled baseline accelerometry from all 14 and 13 entrants, respectively [31,46,47]. Wong et al. enrolled seven participants in an individualized balance training case series and analysed six completers [27]. In Lohi et al. (n = 11), body side rather than participants was randomized: one arm and the contralateral leg were trained, with the opposite limbs serving as controls. Possible cross-education and a largely unachievable elbow-extension prescription limited inference from this limb-level design [65].

The acute experiment with rhythmic auditory stimulation randomized the order of stimulated and standard six-minute walk tests within participants, not the cueing dose. The first 18 participants received stimulation at 100% of cadence, and the next 30 received 110% after interim observations. In the sequential cohort tested at 110%, stimulation increased six-minute walk distance by 8.3 m (95% confidence interval: 1.82-14.78; p = 0.01). This small effect of gait cueing, observed during a single visit, was of uncertain clinical importance and did not represent longitudinal conditioning [66].

Respiratory Muscle Training

Studies of respiratory muscle training evaluated inspiratory training with a pressure threshold, combined inspiratory and expiratory training, and endurance training with normocapnic hyperpnoea at widely varying doses. In Fregonezi et al. (n = 27), between-group results favoured training for maximal inspiratory and expiratory pressures. The full report, however, showed no significant difference between groups in respiratory endurance, only a change in MVV within the training group, and no meaningful benefit in spirometry or lung volumes. Sequence generation and concealment were unreported, withdrawal timing was unclear, effort influenced testing, adherence was unquantified, and the isolated favourable result for the role-physical domain of SF-36 was selected from nine domains [67]. Hsu et al. compared volunteers undertaking training with matched patients unwilling to train [22].

The prescribed programme differed between sections, and information on adherence and medication changes was unavailable. The paper presented separate tests before and after treatment within each group rather than a valid comparison of changes between groups or a group-by-time interaction. A comparative causal effect was therefore not reliably estimable [22]. Weiner et al. reported uncontrolled changes by severity group. Values for inspiratory pressure in the severe group duplicated forced expiratory volume in one second (FEV1) values; respiratory endurance in the severe group and dyspnoea in the moderate group were also reported inconsistently, so their exact magnitudes were unusable [68].

The Leipzig series comprised a selected short-term uncontrolled episode and a later long-term programme in which allocation depended on participant preference. Nine of 10 participants in the 2011 preliminary report mapped directly to participants labelled TG1-TG9 in the final report, seven final participants had received training in 2007, and one previously trained participant later entered as a control [21,44,45]. Only 10 of 20 selected patients entered the short-term episode.

In the long-term programme, allocation depended on willingness, motivation, available time, and previous experience. Only 12 of 18 trainees and five of six controls completed 13 months. Medication, training, and measurement timing were modified around illness, and respiratory endurance testing required maximal effort and active encouragement. The available result was a comparison of observed data from completers rather than an effect of assignment and was at critical risk of bias. Favourable signals for respiratory strength or endurance therefore remain very uncertain and are not independent replications across these overlapping reports.

Perioperative Rehabilitation and Rehabilitation After Crisis or Hospitalization

These studies addressed surgical recovery rather than chronic MG disease modification. Chen et al. reported a trial of 80 participants conducted around thymectomy, but the sequence and concealment methods, participant flow from screening, adherence, medication changes, and assessor blinding were not described. Hospital stay was listed as an outcome, but no result was reported. More importantly, many reported p-values comparing groups for baseline variables, postoperative pulmonary function, and early ADL were incompatible with the published means, standard deviations, and group sizes; the reported day 1 and day 3 ADL standard deviations were also inconsistent. It is therefore not possible to determine whether the means, dispersions, p-values, or analysis denominators are correct. Six-minute walk distance was null, and exact pulmonary and ADL inference remains unreconciled [69].

Ambrogi and Mineo selected 17 of 44 rehabilitation completers and matched them to 17 historical controls who underwent surgery between 1999 and 2004. Reported categorical p-values for admission to intensive care, major morbidity, and improvement at one year were not reproducible from the published counts; the analysis of time to remission excluded participants who never achieved remission rather than censoring them. Only the comparison of length of stay among matched completers was retained as contextual evidence at serious risk of bias, not as a randomized or contemporaneous effect [20]. Polastri et al. supplied only two uncomplicated early postoperative cases and reported a dyspnoea value above the stated scale maximum [70].

Otaka et al. analysed 473 hospitalizations following myasthenic crisis, although the abstract reported 437. Exposure was the accumulated number of billed rehabilitation units after ventilator weaning, not assignment at a common baseline. Recovery speed could therefore determine subsequent dose while also defining the outcome. The higher-intensity group was older and more impaired, with higher rates of tracheotomy and steroid pulse therapy, longer pre-index ventilation, and worse initial ADL. The conventional adjusted hazard ratio was 0.69 (95% confidence interval: 0.48-0.99), whereas inverse-probability weighting gave 0.79 (0.56-1.12). The result was at critical risk of bias and cannot establish that greater rehabilitation intensity delayed recovery [23]. Chang et al. randomized 60 recently discharged adults and analysed 54 complete cases [71].

The retrospectively registered primary comparison between groups for change in maximal inspiratory pressure was null (p = 0.18) [14,71]. Internally consistent secondary results favoured maximal expiratory pressure, six-minute walk distance, FEV1, and FVC. However, registration occurred almost six years after the trial, six randomized participants were excluded, reported Borg score and percent predicted FEV1 values were internally inconsistent, and the published effect size estimates and purported minimal clinically important differences could not be verified. Positive secondary findings were therefore at high risk of bias and could not override the null primary result [14,71].

Juvenile Myasthenia Gravis

One active comparator trial with blinded assessors randomized 30 stable adolescents to treadmill training with partial body weight support added to a substantial supervised physiotherapy programme or to the same physiotherapy alone [72]. Sequence generation, CleanWeb allocation, blinded assessment, complete follow-up, unchanged medication and activity, and approximately 98% attendance were strengths. However, the University Hospital Medical Information Network (UMIN) registry and publication differed in age range, treatment duration, and blinding description; registered outcomes from repetitive nerve stimulation and MGQOL15 were not reported, while PedsQL fatigue and total scores were substituted [15,72].

Spirometry, strength, and walking were influenced by effort; participants knew assignment, many outcomes were tested, confidence intervals were absent, and several reported standard deviations were implausibly small and could not be verified as SD rather than another dispersion measure. The trial suggests a possible incremental benefit from treadmill training but does not support a broad conclusion about the efficacy of juvenile rehabilitation

Disease Stability, Medication, and Pyridostigmine Timing

Stability definitions varied across studies. MGEX and Mohamed et al. required at least six months of stability. The RESTOREX parent report required three months, whereas its linked biomarker report required one month. Ceren et al. excluded recent crisis or a change in treatment dose within one month, and Fregonezi et al. excluded crisis within two months. Chang et al. excluded medication adjustment, unstable symptoms, intravenous immunoglobulin, or plasma exchange within six months [28,29,43,63,67,72,73]. Reports using only the terms controlled, stable, optimized, or outpatient were rated unclear.

MG treatment generally continued, but changes and concurrent immunotherapy were incompletely reported. Mohamed et al., Fregonezi et al., and Wong et al. required unchanged treatment; RESTOREX measured dose changes; Ambrogi and Mineo allowed reduction or withdrawal when symptom-free; other reports did not report changes [20,27,28,67,72].

Rahbek et al. tested performance exactly one hour after the regular pyridostigmine dose [62]. Anggarini et al. scheduled exercise 1.5-2 hours after dosing and stated that intervention and outcome measurement should occur within this peak window; actual test timing was unreported [16]. Mohamed et al. began exercise 1.5-2 hours after dosing and assessed outcomes approximately three hours after ingestion, matching visits where possible [72]. Andersen's journal report specified testing 0.5-2 hours after dosing; compliance was unreported [66]. Wong et al. assessed anticholinesterase users approximately three hours after ingestion, whereas Ambrogi and Mineo scheduled the resistive exercise component at peak effect, usually 1.5-2 hours after dosing [20,27].

RESTOREX tested participants between 12:00 and 14:00. Eighteen participants had taken pyridostigmine six to seven hours earlier, and 20 had taken it two to three hours earlier. Timing was not associated with improvement on the 15-item Myasthenia Gravis Quality of Life Scale (MGQOL15; p = 1.00), and doses could change [28]. No trial randomized timing; these observations do not establish effect modification or an optimal interval.

Adherence, Tolerability, and Adverse Events

Adherence was measured inconsistently through diaries, wearable data, telephone calls, attendance, or supervised visits; other reports provided only qualitative tolerability statements. DIG-MG reported coaching attendance and ring wear, MGEX reported completed sessions, Muslihah et al. reported a mean of 23.8 of 24 sessions among 11 intervention participants, and RESTOREX reported compliance by group [28,40,43,61]. Kartika et al. reported 24 of 24 sessions for all 17 completers (nine in the intervention group and eight in the control group), but not for all randomized participants; Anggarini et al. reported attrition but not attendance at individual sessions [16,42]. These measures were not directly comparable.

Serious events were infrequent but incompletely ascertained, and minor symptoms were variably reported. Both Rahbek training groups reported transient fatigue or bulbar worsening; one participant in resistance training discontinued after substantial bulbar symptoms requiring prednisolone treatment, with causality considered possible but uncertain [62]. MGEX reported no exacerbation in the exercise group and two exacerbation-related hospitalizations in the usual-care group; at least one patient-reported event occurred in 16 of 23 exercise participants and 14 of 20 usual-care participants [43]. RESTOREX reported a crisis in the control group attributed to medication default and a renal event in the exercise group considered unrelated to exercise [28].

Anggarini et al. reported discomfort and intercurrent illness in the intervention group, including sinusitis, hospitalization, and a later death judged unrelated to the intervention; five of 23 randomized participants were not analysed [16]. Farrugia et al. reported no clinically significant relapse, although one participant described an intersession "MG crisis"; no medical intervention or medication change occurred, and Farrugia et al. considered the episode probably a panic attack [64]. Wong et al. reported one withdrawal related to work and one arrhythmia after intervention but before follow-up [27]. Reports without systematic surveillance were not treated as proof of safety.

Complete study characteristics, decisions about links between reports, medication information, adherence, withdrawals, adverse events, funding, and conflicts of interest are provided in Table 1.

**Table 1:** Characteristics of 38 study units: design, participants, interventions/exposures, outcomes, treatment, safety, linkage, data issues, funding, and conflicts of interest. Linked baseline analyses reuse participants from parent intervention cohorts and are not independent cohorts. Unless stated, medication changes and dose/test timing were not reported. In observational studies, adherence and intervention-related adverse events were not applicable; attrition was reported when relevant. Abbreviations: AChEI, acetylcholinesterase inhibitor; CMAP, compound muscle action potential; DOR, delta-opioid receptor; EMT, expiratory muscle training; FSS, Fatigue Severity Scale; HADS, Hospital Anxiety and Depression Scale; HRmax, maximal heart rate; IMT, inspiratory muscle training; IPAQ, International Physical Activity Questionnaire; IVIG, intravenous immunoglobulin; ITT, intention-to-treat; MEP/PEmax, maximal expiratory pressure; MFIS, Modified Fatigue Impact Scale; MG, myasthenia gravis; MG-ADL, MG Activities of Daily Living; MGC, MG Composite; MGFA, MG Foundation of America; MGQOL15/MG-QoL15, 15-item MG quality-of-life scale; MGQOL15F-r, revised French MGQOL15; MIP/PImax, maximal inspiratory pressure; MMS, Myasthenia Muscle Score; MOR, mu-opioid receptor; MVPA, moderate-to-vigorous physical activity; MVV, maximal voluntary ventilation; Neuro-QoL, Quality of Life in Neurological Disorders; PA, physical activity; PBWSTT, partial body-weight-supported treadmill training; PedsQL, Pediatric Quality of Life Inventory; PEF/PEFR, peak expiratory flow/rate; PLEX, plasma exchange; PSQI, Pittsburgh Sleep Quality Index; QMG, Quantitative MG score; RCT, randomized controlled trial; RMET, respiratory muscle endurance training; RMT, respiratory muscle training; sEMG, surface electromyography; SGPALS, Saltin-Grimby Physical Activity Level Scale; Tlim, time to exhaustion; TUG, Timed Up and Go; UMIN, University Hospital Medical Information Network; VAFS, Visual Analogue Fatigue Scale; Vlim, ventilated volume during endurance testing; VO2max/VO2peak, maximal/peak oxygen uptake; Walk-12G, 12-item Generic Walking Scale; NR, not reported; NA, not applicable.

Report citation(s), study design, and context	Participants and disease status	Intervention or exposure and comparator	Follow-up and outcomes	Treatment, timing, adherence, withdrawals, and adverse events	Report linkage, data issues, funding, and conflicts of interest
Report citation(s): Norling et al. [61] (S01). Design/context: nationwide fully digital three-arm RCT, Sweden	81 randomized: PA = 26, sleep = 28, control = 27; 72 completed: PA = 25, sleep = 22, control = 25. Confirmed ocular or generalized MG with disease duration ≥6 months; the publication did not operationalize a separate clinical stability criterion. Among completers: ocular = 5, generalized = 62, asymptomatic = 5	Six-week OURA baseline, then seven remote coaching sessions on physical activity targeting ≥150 min per week of moderate activity and interruption of sitting; comparators: sleep hygiene programme and passive OURA observation	24 weeks: 6-week baseline, 12-week intervention, 6-week follow-up; primary MG-ADL; FSS, Chalder Fatigue Scale (CFS), device-measured physical activity and sedentary time, sleep	Medication: baseline treatment reported; subsequent changes, rescue therapy, and exacerbations were NR. Timing: NR. Adherence: all seven sessions were attended by 68% of the PA group and 82% of the sleep group; ring wear was about 23 h per day among retained participants. Withdrawals: 9/81, by group 1/26, 6/28, 2/27. Adverse events: no systematic method or counts	Report linkage: single report; no overlap confirmed by the source with S10 or S25. Data issues: 81 participants were randomized, but the principal analysis included 72 completers; FSS maxima exceeded the 9-63 scale; sleep, medication, and other reported values were internally inconsistent. Funding: Erling-Persson Foundation and Majbritt Lundberg family donation. Conflicts of interest: Punga reported honoraria or consulting fees; others reported none
Report citation(s): Birnbaum et al. [60] (S02). Design/context: nationwide online cross-sectional survey, France; NCT05408702	455 analysed: 244 current exercisers, 211 non-exercisers; generalized = 363, ocular = 38, asymptomatic = 54; stable = 240, fluctuating = 215. Diagnosis and stability self-reported	Current structured exercise recorded as a binary exposure (yes or no); barriers and facilitators; exercisers versus non-exercisers	Single assessment, November 2021-May 2022; barriers and facilitators, MG-ADL, MGQOL15F-r, Neuro-QoL fatigue	Medication: AChEI = 371, immunosuppressant = 200, corticosteroid = 158, IVIG = 78; changes and timing of dosing relative to sessions were NR. Adherence, withdrawals, and adverse events: NA, observational study	Report linkage: single report. Data issues: one Results sentence reversed the coherent 244/211 exerciser proportions. The Neuro-QoL fatigue categories totalled 495 despite a stated n = 435; the counts in the severe fatigue category totalled 47 rather than the reported 83; and reported percentages were incompatible with the stated denominators. Funding: AFM-Téléthon. Conflicts of interest: none reported
Report citation(s): Kartika et al. [42] (S03). Design/context: outpatient parallel RCT, Surabaya, Indonesia	30 screened; 20 randomized 10/10; 17 analysed 9/8. Age = 18-59 years; analysed participants had generalized MG, MGFA I–IIb; stability period NR	Low-intensity cycle ergometer for 30 min, three times per week for 8 weeks plus breathing education; comparator: breathing education alone	8 weeks; further follow-up was NR; maximum walking speed, cadence, FSS	Medication: baseline pyridostigmine; standard treatment continued; changes were NR. Timing: NR. Adherence: all 17 completers completed 24/24 sessions. Withdrawals: 3/20; interventional group = 1 and control group = 2. Adverse events: no deterioration or training discomfort reported among completers; active surveillance was NR	Report linkage: different ethics approvals and nonidentical flows prevented formal merging with Anggarini [16], but probable partial or substantial overlap could not be excluded: both final intervention groups had n = 9, mean age = 47.78±5.36 years, and 2 men and 7 women. Data issues: the reason for withdrawal and reporting of the FSS and statistical analyses conflicted across source sections. Funding: none. Conflicts of interest: none reported
Report citation(s): Chang et al. [71] and ClinicalTrials.gov record [14] (S04). Design/context: parallel RCT after hospitalization with blinded assessors, Taiwan	68 assessed; 60 randomized 30/30; 54 analysed 26/28. MGFA IIa–IIIb after discharge for acute exacerbation; recovering rather than stable outpatient population	Home-based IMT progressing from 30% to 45% of MIP plus walking for 30 min per day at 50%–70% HRmax and breathing control; comparator: standard care after discharge	6 weeks; retrospectively registered primary MIP (between-group change null, p = 0.18); secondary MEP, 6MWD, forced vital capacity (FVC) and other pulmonary function outcomes, and Borg scores	Medication: use of pyridostigmine, corticosteroids, and azathioprine was reported; doses, group distribution, and actual medication changes after discharge were NR. Timing: NR. Adherence: diaries, calls, and reminders planned; achieved dose was NR. Withdrawals: 6/60. Adverse events: none self-reported; ascertainment NR	Report linkage: single report; no participant overlap with S21 was established. Data issues: trial conducted in 2018 but first registered in October 2024; reported Borg score and percent predicted FEV1 values were internally inconsistent; published effect size estimates and minimal clinically important differences could not be verified and were excluded; primary MIP comparison was null. Funding: stated not applicable. Conflicts of interest: none reported
Report citation(s): Dimmick et al. [55] (S05). Design/context: prospective free-living cohort, single centre, Canada	62 approached; 44 met the inclusion criteria; 30 enrolled; 25 completed; 16 met the requirement of at least 56 usable days and entered final analyses. Historically generalized MG and independently ambulant; ocular-only MG excluded; enrolment MGFA 0–IIIa; stability NR	Continuous wrist ActiGraph plus daily electronic fatigue and percent-normal symptom ratings; no assigned comparator; same-day analyses and participant-specific within-person models	12 weeks; fatigue, symptoms, MG-ADL, MG-QoL, physical activity, inactivity, sleep, and their associations	Medication: obtained from records; IVIG day modelled; other changes and timing of dosing relative to sessions were NR. Adherence, withdrawals, and adverse events: NA, observational study	Report linkage: single report. Data issues: only 16/30 enrolled participants were analysed; daily slider validation was unpublished; inactivity was reported as 702.8±1756.0 min despite a range of 139.7-883.4; extensive exploratory models had no multiplicity correction. Funding: NSERC CREATE We-TRAC, CNDR, NSERC Vanier, Alberta Innovates, University of Calgary Eyes High. Conflicts of interest: none reported
Report citation(s): Alsop et al. [52,53] (S06). Design/context: online survey and volunteer interviews, Australia and other countries	Survey: 85 complete respondents; generalized = 82, ocular = 3; self-reported stability ≥4 weeks. Qualitative companion: 10 selected participants from the same project, all generalized MG, independently mobile, and 8/10 meeting activity recommendations	Seven-day PA and sedentary behaviour survey with physical activity and sitting-time strata; no assigned intervention. Linked interviews examined barriers, facilitators, and professional advice	Survey May-September 2019; MFIS, MGQOL15, Walk-12G, balance confidence; later qualitative assessment of barriers and professional advice	Medication: previous or current anticholinesterase treatment = 77, immunosuppressant = 70, IVIG = 57; changes and timing of dosing relative to sessions were NR. Adherence, withdrawals, and adverse events: NA, observational study	Report linkage: the qualitative report is a selected 10/85 subset of the earlier survey project, not an independent cohort. Data issues: several reported regression estimates contained incompatible coefficients, confidence intervals, or p-values. Funding: none; MG Association of Queensland assisted recruitment. Conflicts of interest: none reported
Report citation(s): Glaubitz et al. [59] (S07). Design/context: prospectively recruited cross-sectional survey, single centre, Germany; DRKS00033171	84 analysed; screened denominator NR. Generalized = 76, ocular = 8; mean QMG = 8.5; no stability restriction	Unvalidated exercise questionnaire covering frequency, duration, and a modified study-specific metabolic equivalent of task (MET) score, and self-reported walking distance; physical activity categories and correlation analyses; no assigned comparator	Single assessment, February 2022-February 2024; physical activity and barriers, QMG, MG-ADL, QoL, correlations with self-reported walking distance	Medication: pyridostigmine = 84/84, corticosteroids = 53, other immunotherapies; changes and timing of dosing relative to sessions were NR. Adherence, withdrawals, and adverse events: NA, observational study	Report linkage: single MG-specific report from the Sport NMD study. Data issues: recruitment began in February 2022, but registration occurred in December 2023; nonstandard study-specific MET score; MG-ADL was reported inconsistently as 8.4 and 4.8; exercise duration totals did not equal the stated denominator; one confidence interval included zero despite p = 0.005; walking distance was inconsistently called step count. Funding: no direct study funding; Projekt DEAL supported open access. Conflicts of interest: several commercial relationships disclosed
Report citation(s): Steyaert et al. [56] and ClinicalTrials.gov record [12] (S08). Design/context: prospective decentralized digital cohort, USA; NCT04590716	531 interested persons identified; 443 approved; 232 downloaded or registered; 113 onboarded; 82 completed. Diagnosis documentation was requested after enrolment, but the verified proportion was not reported. Forty-five completers reported an exacerbation. The step-count versus MG-ADL analysis included 26 participants, and the exacerbation-status versus step-count analysis included 14	Daily electronic MG-ADL scores and self-reported exacerbation status recorded by smartphone, plus optional platform-derived step counts; no assigned comparator	3 months; exacerbations, daily MG-ADL, symptom clusters, associations of step count with MG-ADL and exacerbation status	Medication: active MG medication use was reported inconsistently; 28% reported treatment for refractory disease; changes and timing of dosing relative to sessions were NR. Adherence: not applicable because no intervention was assigned. Withdrawals: 31/113 did not complete observation, derived from 113 onboarded and 82 completed. Adverse events: not applicable because no intervention was assigned	Report linkage: single report. Data issues: strong recruitment and adherence selection; step counts obtained from Apple HealthKit, Google Fit, or Validic without a common wear time protocol; the number of exacerbation days was reported as both 525 and 526; multiple nested denominators. Funding: UCB Pharma; Sharecare participated in sponsorship, study design, and data activities. Conflicts of interest: multiple authors were employees or stockholders of UCB or Sharecare
Report citation(s): Misra et al. [28], Tripathi et al. [29], and CTRI record [13] (S09). Design/context: open-label single-centre RCT, India	40 randomized 20/20; 38 parent trial completers analysed 19/19. Generalized MGFA II–III. The linked report selected 15 walking participants plus 12 healthy baseline controls; exact selection from the 19 completers could not be reconciled, and sex and stability descriptions differed between reports	Daily walking progressing from 10 to 20 and then 30 min; comparator: 30 min daily rest	3 months; follow-up beyond intervention NR; MGQOL15 response, 6MWD and step count, MG-ADL, MMS; beta-endorphin, MOR, and DOR biomarkers in the subset	Medication: median pyridostigmine 240 mg/day, prednisone 15 mg/day; dose changes were allowed and analysed. Timing: testing 2-3 or 6-7 h after dosing; subset NR. Adherence: parent walking arm = 97.10 ± 4.1%, and rest arm = 97.5 ± 3.7%; in the 15-person subset, 9 had 100%, 2 had 89%, and 4 had 90%-94% compliance. Withdrawals: one participant in each group. Adverse events: renal event in exercise arm; medication-default crisis in control; systematic subset count was NR	Report linkage: Tripathi is a selected walking group subanalysis, not a second trial; healthy controls informed baseline biology only. Data issues: parent report stated 19/19 MGQOL15 responders versus 6/15 in the linked subset; one reported median lay outside its own IQR; P-values for medication doses were incompatible with reported means, SDs, and sample sizes. Funding: parent World Federation of Neurology India Trust; subset NR. Conflicts of interest: none reported
Report citation(s): Punga et al. [26] (S10). Design/context: prospective uncontrolled community-gym case series, Sweden	13 invited; 7 enrolled, completed the intervention, and were analysed. Mild-to-moderate generalized MG; all MGFA II; described as well controlled; formal stability duration was NR	Tailored aerobic, resistance, and balance training, two 60-min sessions per week, with at least one supervised; no comparator	6 months; individualized training load progression, rowing distance and load, photographic and video observations, FSS, MG-QoL15, and feasibility and tolerability; no standardized test of aerobic capacity or balance	Medication: unchanged; no additional post-exercise pyridostigmine. Timing: standardized interval was NR. Adherence: 83.5%-98%; the source described this anomalously as median 93 ± 5%. Withdrawals: 0/7 after enrolment. Adverse events: transient neck weakness or fatigue in 2, soreness in 1, one participant stopped spinning, stress-associated flare in 1	Report linkage: participants were recruited mainly from S25; exact overlap was not reported. The source did not confirm overlap with the DIG-MG (Digital Phenotyping and Lifestyle Intervention in Patients With Myasthenia Gravis) cohort (S01). Data issues: “all improved” did not reflect mixed FSS and MG-QoL15 trajectories; “median 93±5%” and prednisone 5 versus 10 mg were inconsistent. Funding: Selanders Foundation, Neuroförbundet, ALF, Swedish Research Council. Conflicts of interest: none reported
Report citation(s): Chen et al. [69] (S11). Design/context: reported parallel perioperative RCT in patients undergoing thymectomy, China	80 reportedly enrolled, randomized, and analysed (40 per group); participant flow from screening was NR. Adults <65 years undergoing extended thymectomy; MGFA I–IIb; stability was NR	Five preoperative days of treadmill walking or stair climbing, inspiratory training, and chest physiotherapy, followed by postoperative respiratory and resistance rehabilitation; comparator: chest physiotherapy, deep-inhalation training, routine medication, and nursing care	Preoperative days 1-5 and postoperative days 1-5; pulmonary function testing (PFT) and 6MWD on postoperative day 7; activities of daily living (ADL) on days 1, 3, 5; hospital stay prespecified but unreported	Medication and timing: perioperative MG regimen, changes, and timing NR. Adherence: NR. Withdrawals: reported results imply that all 80 were analysed; participant flow NR. Adverse events: no crisis or linked side effects reported; definitions, surveillance methods, and counts were NR	Report linkage: single report. Data issues: the method of randomization, allocation concealment, and participant flow from screening were NR; sample size arithmetic (“loss rate 120%”) uninterpretable; hospital stay prespecified but unreported; many baseline, PFT, and ADL p-values could not be reproduced from reported means, SDs, and sample sizes; ADL SDs on days 1 and 3 conflicted. Exact inferential results require author clarification. Funding: no grants. Conflicts of interest: none reported
Report citation(s): Mohamed et al. [72] and UMIN record [15] (S12). Design/context: parallel outpatient RCT with blinded assessors, Egypt; UMIN000044102	30 randomized, analysed, and completed the study (15 per group). Age = 13–16 years; anti-AChR-positive generalized juvenile MG, MGFA IIa/IIb; symptoms stable ≥6 months by QMG	PBWSTT 30 min added to active 60-min physiotherapy with breathing, resistance, balance, and stretching three times per week; comparator: active physiotherapy alone	12 weeks; FVC, forced expiratory volume in one second (FEV1), PEFR, MVV, CMAP, and strength, postural stability, 6MWD, PedsQL fatigue and quality of life	Medication: all used pyridostigmine; medication and habitual activity were required to remain unchanged. Timing: exercise 1.5-2 h and testing about 3 h after dosing. Adherence: about 98%. Withdrawals: 0/30. Adverse events: diaries captured symptoms; exercise paused for pain or exhaustion; no exacerbations; number of mild events was NR	Report linkage: single report. Data issues: registry and publication differed in age range, physiotherapy duration, and blinding; registered RNS and MGQOL15 were unreported, and PedsQL was reported instead; several reported SDs were exceptionally small and could not be verified. In the control arm, PEFR increased from 165.46 to 171.80 L/min, and the reported change was +6.34 L/min (3.83%). Funding: no external funding. Conflicts of interest: none reported
Report citation(s): Andersen et al. [66] (S13). Design/context: acute within-participant comparison of two 6MWTs with randomized test order, single visit, Denmark	48 analysed; numbers screened and enrolled = NR. Generalized MG; median MGC = 7.5; observed MGFA distribution and formal stability criterion were NR	Walking with rhythmic auditory stimulation: the first 18 participants were tested at 100% and the next 30 at 110% of their fastest cadence; comparator: standard 6MWT without stimulation	One visit; tests 30 min apart; no follow-up; 6MWD, walking speed, heart rate and Borg rating	Medication: pyridostigmine and immunosuppression reported qualitatively; counts and changes NR. Timing: protocol 0.5-2 h after dosing; actual compliance NR. Adherence: not applicable to a single acute exposure; synchronization was not measured. Withdrawals: none reported. Adverse events: systematic ascertainment of falls, symptom worsening, and crisis was NR	Report linkage: participant overlap with the S15 clinic component was not reported and was not assumed. Data issues: randomized test order but not cueing dose; only 30 min separated maximal tests; cadence changed from 100% to 110% and sample size was adapted after interim inspection. Funding: Rigshospitalet and Grosserer L. F. Foghts Fond. Conflicts of interest: none reported
Report citation(s): Andersen et al. [50] and Andersen and Vissing [51] (S14). Design/context: web survey of people identified through the registry, Denmark	Parent cohort: 1,745 people identified through the registry, 779 respondents, 662 complete plus 117 partial; SGPALS, n = 731. The 2021 adjusted complete-case model of the association between SGPALS and fatigue used n = 690; the linked 2022 IPAQ complete-case analysis used n = 691. Adults identified through a registry code who also self-identified as having MG; no individual examination or stability restriction	Self-reported SGPALS activity over the prior year and IPAQ sitting, walking, and MVPA over the prior 7 days; activity and disease-severity strata; no assigned intervention	One survey, June-November 2019; fatigue, MG-ADL, MG-QOL15, sitting, walking, MVPA	Medication: pyridostigmine 448 (reported as 56%; overall survey, n = 779) in 2021 and 399/691 in 2022; immunosuppressive and other treatment recorded; changes and timing of dosing relative to sessions were NR. Adherence, withdrawals, and adverse events: NA, observational study	Report linkage: both reports analyse the same national survey; the 2022 report additionally contains S15. Data issues: response rate used a model-based eligibility estimate; the source reported pyridostigmine as 448 (56%) despite the overall n=779; at least two adjusted coefficients lay outside their reported confidence intervals. Funding: 2021 NR; 2022 Rigshospitalet Research Foundation. Conflicts of interest: none reported
Report citation(s): Andersen and Vissing [51] (S15). Design/context: cross-sectional device study, Denmark	69 clinic participants supplied ActiGraph data; MG-ADL missing for 5 and IPAQ for 6; screening, invitation, refusal, and exclusion denominators were NR. Verified adults with MG; historical MGFA II–IV; median current MG-ADL 2; stability was NR	Waist ActiGraph for 7 days plus IPAQ; disease-severity and activity strata; no assigned comparator	Seven-day measurement; no follow-up; sedentary time, physical activity intensity, step count, and associations with MG-ADL	Medication: pyridostigmine = 50/69; immunosuppression = 53/69; changes and timing of dosing relative to sessions were NR. Adherence, withdrawals, and adverse events: NA, observational study	Report linkage: separate clinic component within Andersen and Vissing [51]; recruitment overlapped in time with S14, but participant overlap was not reported. Data issues: several reported regression values had impossible intercepts, confidence intervals, or p-values; main coefficients for MG-ADL were internally coherent. Funding: Rigshospitalet Research Foundation. Conflicts of interest: none reported
Report citation(s): Otaka et al. [23] (S16). Design/context: retrospective nationwide administrative cohort, Japan	14,231 MG admissions identified; 652 mechanically ventilated without thymectomy; 473 analysed: 234 in the group with more accumulated billed rehabilitation and 239 in the group with less. Rehabilitation began after ventilator weaning following myasthenic crisis; subtype, MGFA class, and stability were NR	Higher versus lower accumulated billed inpatient rehabilitation units per day after ventilator weaning; no baseline assignment to an intensity strategy; rehabilitation content, actual participation, and information on tailoring based on fatigability were unavailable; 91% received some rehabilitation	Primary outcome: recovery to an ADL score of 0. Secondary outcomes: recovery to an ADL score of 1 and to no more than 50% of the index ADL score; deaths were censored and reported	Medication: PLEX 236, IVIG 353, steroid pulse therapy 338; maintenance changes and timing of dosing relative to sessions unavailable. Adherence: billed units, not participation or fidelity. Withdrawals: 15% of outcome data were missing and imputed. Adverse events: in-hospital deaths 1/234 versus 7/239, not adjudicated in relation to therapy; falls, crisis, respiratory events, and session events not captured	Report linkage: single report. Data issues: abstract n=437 versus verified n=473; rehabilitation exposure was measured after ventilator weaning and could depend on recovery, creating critical concern about confounding and reverse causation; conventional hazard ratio 0.69 (0.48-0.99) versus weighted hazard ratio 0.79 (0.56-1.12). Funding: Japanese Ministry of Health grant 20AA2005. Conflicts of interest: Otaka was a Novartis employee; others: none reported
Report citation(s): Ceren et al. [73] (S17). Design/context: randomized crossover trial with blinded assessors, single centre, Turkey; NCT03743740	17 assessed; 10 randomized 5/5 and completed both periods. Adults 18-65 years with generalized MGFA II–III; no crisis or treatment change in the prior month	Published contrast: supervised spinal stabilization exercise versus home breathing exercises and calisthenics. Registry contrast: spinal stabilization plus home programme versus home programme alone; exact contrast unresolved	Two 6-week periods with 4-week washout; FSS and VAFS, dynamometry, PFT, 6MWT, QMG, MGQOL-15	Medication: names and doses were NR; no change from the prior month; during-trial changes NR. Timing: NR. Adherence: supervised session and home-programme adherence NR. Withdrawals: 0/10. Adverse events: none reported; predefined surveillance method and severity-specific event reporting were NR	Report linkage: single report. Data issues: mismatch between the registry and publication descriptions of the comparison; period, sequence, and carryover unmodelled; unpaired analysis of crossover data; post hoc stopping; the reported change in left elbow extension of 8.76 was inconsistent with the reported values before and after treatment. Funding: none. Conflicts of interest: none reported
Report citation(s): Chang et al. [63] (S18). Design/context: prospective uncontrolled cohort recruited from community and outpatient settings, Taiwan	35 included, 34 completed; QMG and QoL n = 32. AChR-positive MGFA II, n = 21, and III, n = 14; no medication adjustment for 6 months; unstable MG or recent IVIG or PLEX excluded	Individualized aerobic and body-weight resistance programme with monthly supervised visits plus home training; no comparator; post hoc groups based on exercise dose	24 weeks; QMG, QoL, grip, FVC, walking speed, body composition. Overall QMG improved from before to after the programme, but the post hoc interaction comparing higher and lower self-selected exercise doses was not significant for QMG (p = 0.165) or quality of life (QoL) (p = 0.739)	Medication: corticosteroids = 14, other immunosuppressants = 10; medication changes during follow-up and AChEI dose were NR. Timing: NR. Adherence: self-reported; reported exercise-dose calculations were inconsistent. Withdrawals: 1/35, reason and timing NR; outcome, n = 32. Adverse events: no negative effects reported; prespecified surveillance methods and counts were NR	Report linkage: single report; no participant overlap with S04 or S21 was established. Data issues: denominators varied across analyses (35, 34, and 32); self-selected exercise dose and post hoc median split confounded by baseline ability; no demonstrated dose-response for QMG or QoL; baseline QMG was reported once as 10.47% although QMG is a score; sarcopenia prevalence was reported inconsistently as 8% and as 8/35 (22.9%). Funding: Shin Kong Wu Ho-Su Memorial Hospital. Conflicts of interest: none reported
Report citation(s): Birnbaum et al. [30,43] (S19). Design/context: multicentre parallel RCT, France; NCT02066519	43 randomized: exercise = 23, usual care = 20; ITT = 43; 41 completed. Adults with stable generalized autoimmune MGFA II–III. Linked baseline accelerometry: 41 returned recordings, 5 failed wear criteria, 3 male recordings were excluded, leaving 33 women; 66 external controls were matched 2:1 by age, height, and weight	Home rowing for 40 min, three times per week for 12 weeks versus usual care with no prescribed increase in exercise; linked baseline DynaPort activity comparison	Three-month observation, 12-week intervention, 3-month follow-up after the programme; MGQOL15F primary; MG-ADL, 6MWD, strength, PFT, fatigue, device-measured physical activity and sedentary time	Medication: AChEI = 37/43, corticosteroids = 18, other immunosuppressants = 28; monthly checks, no difference between groups. Timing: NR. Adherence: 16/23 adherent; mean = 24 sessions. Withdrawals: two participants did not complete the study; all 43 were retained in the intention-to-treat analysis, but the detailed flow was inconsistent. Adverse events (exercise versus usual care): exacerbations, 0 versus 3 by month 6 and 0 versus 5 by month 9; hospitalizations, 0 versus 2; no deaths or life-threatening or disabling events	Report linkage: baseline accelerometry and RCT are one MGEX family, not independent studies. Data issues: 45 enrolled versus 43 randomized; the linked report had 36 valid recordings and excluded three men, leaving 33 women, whereas the RCT reported accelerometer data for 37, leaving one female recording unreconciled after accounting for the three men; completed-session range reached 38 despite 36 prescribed; confidence interval for knee extension and p-value conflicted. Funding: Assistance Publique–Hôpitaux de Paris, French PHRC, Institute of Myology. Conflicts of interest: none relevant reported
Report citation(s): Ruiter et al. [57] (S20). Design/context: online cross-sectional survey drawn from a Dutch-Belgian MG registry, Netherlands	558 invited, 420 analysed; multivariable n = 397. Autoimmune MG; AChR about 80% among those with known results; MGFA class and stability were NR	Self-reported minutes of moderate physical activity per day and weekly sessions of strenuous exercise; activity strata; no assigned intervention	Single survey; fatigue, MG-ADL, MGQOL15, HADS, PSQI, associations between physical activity and fatigue	Medication: corticosteroid, immunosuppressive, and pyridostigmine use collected; changes and timing of dosing relative to sessions were NR. Adherence, withdrawals, and adverse events: NA, observational study	Report linkage: single report. Data issues: 23 excluded from the adjusted model; registry participation was voluntary; physical activity was crudely self-reported; ascertainment of non-autoimmune comorbidities was not systematic; the BMI standard deviation was reported inconsistently as 5.0 and 1.0. Funding: no specific grant. Conflicts of interest: Ruiter reported none; Tannemaat and Verschuuren disclosed institutional and industry relationships
Report citation(s): Hsu et al. [22] (S21). Design/context: prospective non-randomized controlled cohort, single centre, Taiwan	40 recruited; 6 excluded at unclear stage; 34 analysed: RMT = 18, disease control = 16. Ambulatory generalized MGFA II–III; stability interval was NR	Home-based threshold RMT plus usual treatment; comparator: matched patients who were unwilling to undertake RMT and received usual treatment, not a randomized or preference-neutral control	12 weeks; PFT, MIP, MEP, 6MWD, QMG, MGC, and fatigue. Only separate tests before and after treatment within each group were presented; no valid group-by-time interaction or between-group difference in change was reported	Medication: adjunctive conventional treatment; regimens and changes by group were NR. Timing: NR. Adherence: weekly calls; dose and session count were NR; the prescribed frequency differed between report sections. Withdrawals: all 34 apparently analysed. Adverse events: none reported in RMT; predefined adverse-event categories, counts, and safety data for the control group were NR	Report linkage: single report; no participant overlap with S04 was established. Data issues: training frequency conflicted across abstract, Methods, and Discussion; adherence and medication changes unavailable; comparative effect not reliably estimable. Funding: Chang Gung Memorial Hospital grants. Conflicts of interest: no commercial associations reported
Report citation(s): Farrugia et al. [64] (S22). Design/context: prospective uncontrolled rehabilitation pilot, UK	25 approached, 10 enrolled, 9 with post-baseline data. Clinically stable MG with disabling fatigue; MGFA was NR	Weekly 2-h programme combining paced aerobic, resistance, and flexibility exercises with breathing exercises, relaxation, and psychological fatigue management; no comparator	10 weeks plus 3-month follow-up; MGC, MG-ADL, MG-QoL, MFIS, FSS, and VAFS (0 = worst, 10 = normal). VAFS improved at week 10 but was not sustained; repeated observations were analysed with an unpaired test	Medication: unchanged during programme. Timing: NR. Adherence: 4/9 missed 1-2 sessions. Withdrawals: 1/10 after first session. Adverse events: no clinically significant relapse was recorded; one participant described an intersession “MG crisis” that required no medical intervention or medication change and was considered by the authors probably a panic attack; predefined adverse-event classification was NR	Report linkage: single report. Data issues: the source described the FSS range as 0-63 although the instrument range is 9-63. Funding: MyAware. Conflicts of interest: none reported
Report citation(s): Rassler et al. [45] and Freitag et al. [21] (S23). Design/context: preference-allocated controlled RMET cohort with overlapping preliminary report, Germany	Final cohort: 24 participants, including 18 in training and 6 in control; 13-month completers: 12/18 and 5/6. Nine of 10 participants in the preliminary report mapped directly to participants labelled TG1-TG9; seven participants in the final cohort had received RMET in 2007, including one who later entered as a control	RMET using normocapnic hyperpnoea: 20 sessions over 4 weeks, then 12-month maintenance; comparator: no RMET and no sham intervention	Total 13 months; respiratory endurance Tlim and Vlim, Besinger score, lung function, physical fitness	Medication: heterogeneous AChEI and immunosuppression; increases with illness or infection. Timing: fixed timing intended; exact interval unconfirmed. Adherence: described as regular except during health-related interruptions; percentage NR. Withdrawals: training = 6/18, control = 1/6. Adverse events: transient neck or arm weakness during testing; serious comorbid events or procedures prompted several withdrawals; relatedness NR	Report linkage: Rassler 2011 is a substantially overlapping preliminary report; 7 final participants previously entered S31, and one previously trained participant later served as control. Data issues: preference allocation, health-related attrition, modifications to medication and training, testing influenced by effort and encouragement, and selective reporting produced critical risk. Funding: Deutsche Myasthenie-Gesellschaft. Conflicts of interest: none reported
Report citation(s): Westerberg et al. [48] and Sabre et al. [49] (S24). Design/context: linked cross-sectional comparisons between countries and between MG and control groups, Sweden and Estonia	Sweden: 70 invited, 40 surveyed, 34 examined; controls 188/400. Estonia: 36/92 in Sabre. Mixed ocular and generalized MG; no uniform stability criterion	Self-reported regular and focused exercise and lifestyle categories; comparisons with population controls, between countries, and among MG subgroups; no assigned intervention	One-time survey and examination; exercise prevalence, fatigue, disease-specific, social, and lifestyle measures	Medication: Swedish cohort AChEI = 27/40, corticosteroids = 9, steroid-sparing = 14; changes and timing of dosing relative to sessions were NR. Adherence, withdrawals, and adverse events: NA, observational study	Report linkage: Ref. [48] and Ref. [49] reuse the same Swedish (n = 40) cohort. Possible overlap with the 13-person Jönköping exercise cohort (S28) could not be reconstructed from the common 70-patient frame and ethics approval. Data issues: one Swedish case lacked a responding matched control; activity denominators varied; reported overweight and obesity values based on BMI were conflicting. Funding: Neuro Sweden, Selanders Foundation, FUTURUM, Swedish Institute, Swedish Research Council. Conflicts of interest: none reported
Report citation(s): O'Connor et al. [31] (pooled analysis of baseline data) and Westerberg et al. [47] (S25). Design/context: prospective uncontrolled exercise cohort, Sweden	54 eligible patients invited; 24 agreed; 8 withdrew before screening; 2 excluded; 14 entered; 11 completed. O'Connor pooled all 14 baseline participants with all 13 S28 entrants. Stable mild-to-moderate MG; completers: AChR-positive = 8, MuSK-positive = 1, seronegative = 2	Supervised cycle intervals, resistance, balance, and stretching, two 90-min sessions per week; no comparator	12 weeks; MGC and QMG, CMAP, muscle force and thickness, chair stand, performance; pooled baseline device measures of physical activity and sedentary behaviour	Medication: mostly stable; 3 reduced cholinesterase-inhibitor dose. Timing: tests at the same time of day; exact interval was NR. Adherence: mean = 88%. Withdrawals: 3/14. Adverse events: no deterioration or discomfort reported; prospective adverse-event classification was NR	Report linkage: The O'Connor sample (n=27) comprised all 14 participants from S25 and all 13 participants from S28; S10 was recruited mainly from S25. Data issues: three completers reduced cholinesterase-inhibitor dose; an increase in MG-QoL15 from 5 to 8 was incorrectly described as improvement, although higher is worse; selected leg-specific outcomes coexisted with multiple null results. Funding: reported in the source publications. Conflicts of interest: reported in the source publications
Report citation(s): Ambrogi and Mineo [20] (S26). Design/context: retrospective propensity-matched thymectomy cohort, Italy	46 consecutive rehabilitation entrants (2005–2010); 2 unable to sustain the programme were excluded; 44 completers; 17 selected completers matched to 17 historical controls who underwent surgery in 1999–2004. AChR-positive MGFA II–III; clinically stable on an unchanged AChEI regimen	Multimodal resistance, aerobic, respiratory, and bulbar exercise 30 min three times per week before and after thymectomy; historical comparator without rehabilitation	3 months preoperative, surgery, 1-month delay, 3 months postoperative; ICU need, morbidity, stay, QMG, fatigue, 6MWT, FVC, and 36-Item Short Form Health Survey (SF-36)	Medication: median AChEI 180 mg; corticosteroids; reduction allowed if symptom-free; actual changes were NR. Timing: resistive exercise usually 1.5-2 h after dosing. Adherence: unquantified; fatigue prompted pauses or modification of the programme. Withdrawals: 2/46 could not sustain programme; 17/44 matched. Adverse events: mild fatigue 18/44, severe pneumonia 1; attribution uncertain	Report linkage: single report. Data issues: historical rather than contemporaneous comparator; only 17/44 completers matched; P-values for ICU admission, major morbidity, and the 1-year assessment were not reproducible from reported counts; time-to-remission analysis excluded non-remitters instead of censoring them. Funding: no external source named. Conflicts of interest: none reported
Report citation(s): Rahbek et al. [62] (S27). Design/context: randomized active comparator feasibility trial, Denmark	15 randomized: aerobic = 8, resistance = 7; 12 completed and were analysed (6 per group). Seropositive generalized MG; MGFA IIa 10, IIb 4, IIIa 1; 4-week run-in assessed stability	Twenty supervised progressive resistance sessions versus 20 supervised cycling sessions for aerobic training	Up to 8 weeks; outcomes assessed 48-120 hours after the final session; feasibility and tolerability, QMG, MG-QoL15, strength, VO2peak, 6MWT and functional outcomes	Medication: pyridostigmine 13, azathioprine 7; intended unchanged; 1 resistance participant started prednisolone. Timing: tests 1 h after dosing. Adherence: among completers, aerobic = 91.7%, resistance = 98.3%. Withdrawals: 3/15. Adverse events: bulbar symptoms = 2, fatigue = 3; one possible intervention-related withdrawal	Report linkage: single report. Funding: Familien Hede Nielsens, Helga & Peter Kornings, Augustinus, Aase & Ejnar Danielsens foundations. Conflicts of interest: none reported
Report citation(s): O'Connor et al. [31] (pooled analysis of baseline data) and Westerberg et al. [46] (S28). Design/context: prospective uncontrolled pilot, Sweden	13 enrolled and were assessed at baseline; 10 completed. Well-regulated MGFA I–II; completers: ocular = 4, generalized = 6; formal stability duration was NR	Supervised aerobic intervals plus resistance and balance, two 75-min sessions per week; no comparator	12 weeks; MGC, PEF, CMAP, 6MWT, chair stand, strength and training loads, and biomarkers; pooled baseline device measures of physical activity and sedentary behaviour	Medication: diverse AChEI and immunotherapies; planned rituximab was assigned to different participant numbers in different parts of the report. Timing: tests at the same time of day; exact interval was NR. Adherence: attendance was 95% for eight completers and 71% for two. Withdrawals: vertebral compression fracture, spinal stenosis, and planned thymectomy. Adverse events: attribution of the reported events and systematic event reporting were NR	Report linkage: The 13 baseline participants reported by O'Connor were pooled with S25; possible participant overlap with the S24 Jönköping survey could not be reconstructed. Data issues: The number of participants scheduled to receive rituximab differed between parts of the report; improvement in training resistance weights is a process measure and not an independent measure of clinical strength. Funding: reported in the source publications. Conflicts of interest: reported in the source publications
Report citation(s): Polastri et al. [70] (S29). Design/context: two-patient postoperative case series, ICU and surgical ward, Italy	2 analysed. AChR-positive generalized MG grade IIa with thymoma; medically optimized; formal stability duration was NR	Immediate post-thymectomy respiratory exercise, mobilization, and incentive spirometry, 20-30 min per day for four sessions; no comparator	Postoperative days 1-4; discharge about day 6; no follow-up after discharge; walking, stairs, Barthel Index, pain, dyspnoea	Medication: patient 1 received pyridostigmine 60 mg four times daily; patient 2 received it three times daily; preoperative PLEX; postoperative changes were NR. Timing: NR. Adherence: both completed four sessions. Withdrawals: 0/2 during admission. Adverse events: no related event or complication reported during hospitalization	Report linkage: single report. Data issues: one dyspnoea visual analog scale entry exceeded the stated scale. Funding: NR. Conflicts of interest: NR
Report citation(s): Wong et al. [27] (S30). Design/context: prospective individualized balance training case series, Australia	7 enrolled; 6 completed intervention; one additional participant missed late follow-up. Physician-confirmed controlled stable MG; generalized 5; detailed MGFA was NR. The report misleadingly described results for seven intervention completers, although only six completed the intervention	Individualized balance, strength, and endurance workstation programme, 16 sessions, one or two per week; repeated baseline, no concurrent control	16 sessions plus 4-week follow-up; QMG, three TUG variants, postural sway, and 6MWT	Medication: unchanged. Timing: tests about 3 h after anticholinesterase dosing. Adherence: 6 completed 16 sessions. Withdrawals: 1 work-related; another missed follow-up after illness and an arrhythmia. Adverse events: none during intervention; arrhythmia not attributed to exercise	Report linkage: single report. Data issues: only five participants had repeated baselines; the percentage-change signs were incorrect for the manual dual-task TUG result in participant 7 and the foam-surface, eyes-closed balance result in participant 5; the authors' 15% threshold for clinically significant improvement was not validated in MG. Funding: MG Association of Queensland. Conflicts of interest: formal statement NR
Report citation(s): Rassler et al. [44] (S31). Design/context: prospective uncontrolled RMET study with detraining, Germany	20 selected; 6 declined participation, 4 could not perform the technique or achieve the prescribed intensity, and 10 entered, completed, and were analysed. Mild-to-moderate generalized MG; Osserman IIa 9, IIb 1; stability duration was NR	Home-based RMET using normocapnic hyperpnoea, with 20 sessions of 30 min each over 4-6 weeks; no comparator	4-6 weeks of training plus 3-5 months of detraining; respiratory endurance Tlim and Vlim, Besinger score, lung function, breathing pattern	Medication: all AChEIs, azathioprine = 7; stable during training; one pyridostigmine reduction during detraining. Timing: fixed timing intended; exact interval was NR. Adherence: 10/10 completed 20 sessions. Withdrawals: none after entry; 10/20 declined participation or could not meet the technique and intensity requirements before cohort entry. Adverse events: no complications or negative effects reported	Report linkage: 7/10 entrants later participated in S23 (six later trainees and one later control), so samples are not independent. Data issues: selected entrants who were technically capable and motivated; repeated testing with the best result retained and active encouragement. Funding: NR. Conflicts of interest: NR
Report citation(s): Fregonezi et al. [67] (S32). Design/context: parallel RCT with blinded assessors, Spain	29 recruited; 2 withdrawals before the programme; 27 completed: training 14, control 13. Stable generalized MG, Osserman IIa or IIb; no crisis in prior 2 months	Eight-week diaphragmatic breathing, interval threshold IMT progressing from 20% to 60% of PImax, and pursed-lip breathing three times per week; comparator: one breathing and energy-conservation session plus standard care	8 weeks; PImax and PEmax favoured training; no significant between-group difference in respiratory endurance; MVV improved only within the training group; spirometry and lung volumes did not differ between groups; one of nine SF-36 domains, role physical, was favourable	Medication: pyridostigmine, azathioprine, prednisone unchanged. Timing: NR. Adherence: called satisfactory; counts were NR. Withdrawals: 2/29, timing relative to randomization unclear. Adverse events: events during the active phase were not tabulated; one crisis before the programme, attribution was NR	Report linkage: single report. Data issues: MG IIa/IIb counts totalled 14 in the n = 13 control arm and 13 in the n = 14 training arm and appear reversed, but the correct allocation could not be confirmed; medication wording was garbled. Funding: CNPq-Brasil fellowship and participation by Red Respira-SEPAR and Instituto de Salud Carlos III. Conflicts of interest: formal statement was NR
Report citation(s): Weiner et al. [68] (S33). Design/context: prospective uncontrolled RMT study stratified by disease severity, Israel	18 enrolled and apparently analysed: moderate = 10, severe = 8. Generalized MG, Osserman IIa, IIb, or III in the moderate group and IV in the severe group; stability duration was NR	Threshold RMT 30 min per day, 6 days per week for 3 months; participants with moderate disease received IMT plus EMT, whereas those with severe disease received IMT only; no comparator	3 months; later follow-up NR; PImax, PEmax, endurance, PFT, blood gases, dyspnoea	Medication: all AChEI; prednisone = 11; two participants in the severe group received supplemental oxygen before training; changes prohibited, verification NR. Timing: NR. Adherence: numeric adherence NR. Withdrawals: participant flow was NR. Adverse events: systematic event reporting was NR	Report linkage: single report; overlap with the unretrieved Gross 1993 study could not be assessed. Data issues: PImax values in the severe group duplicated FEV1; baseline respiratory endurance in the severe group was reported inconsistently; the reported dyspnoea value in the moderate group exceeded the stated scale maximum and conflicted with another value in the report. Exact magnitudes unusable. Funding: NR. Conflicts of interest: NR
Report citation(s): Grohar-Murray et al. [58] (S34). Design/context: cross-sectional 18-state postal survey, USA	550 packets mailed; 250 analysed, an approximately 46% response rate. Self-reported MG; generalized 223, ocular 27; MGFA class and stability were NR	Self-reported aerobic exercise as a self-care action. The report is internally inconsistent: one frequency estimate states that 20% of 250 participants used aerobic exercise (nominally implying 50), whereas the analysis uses 36 exercise users (25 reporting benefit and 11 reporting no benefit). No independent group of non-exercisers was presented	One survey; fatigue scale, functional status, perceived helpfulness of self-care actions	Medication: NR. Timing: NR. Adherence, withdrawals, and adverse events: not applicable to this observational study	Report linkage: single report. Data issues: one reported frequency nominally implied 50 exercise users, whereas the analysis used 36 (25 helpful, 11 not helpful); exercise users were compared with a total sample denominator that included them; totals by functional category were incomplete. Funding: NR. Conflicts of interest: NR
Report citation(s): Lohi et al. [65] (S35). Design/context: randomized within-participant trial with training assigned to one arm and the opposite leg, Sweden	11 enrolled, randomized by limb side, and analysed. Mild-to-moderate MG; MGFA and stability interval were NR	Dynamic strength training of one arm and the opposite leg, 27–30 sessions; comparator: contralateral untrained limbs	10 weeks; maximal isometric strength and fatigability in elbow and knee muscle groups	Medication: AChEI 8/11; changes were NR. Timing: fixed; exact interval was NR. Adherence: only one participant completed the planned elbow-extension training dose; lower-limb prescription was more feasible. Withdrawals: none. Adverse events: initial pain or soreness followed testing; no training-related MG worsening reported	Report linkage: single report. Funding: Swedish and Gothenburg Multiple Sclerosis societies. Conflicts of interest: no commercial benefit reported
Report citation(s): Thomsen et al. [54] (S36). Design/context: prospective population cohort with matched controls, Denmark	MG 107 at baseline and 70 at follow-up; controls 50 and 35. Baseline MGFA: remission 5.6%, I 11.2%, II 60.8%, III 21.5%, IV 0.9%; no stability criterion	Self-reported physical activity at two visits; comparisons with matched controls and within-participant correlations of changes in physical activity and function in the MG group; no assigned training	Mean follow-up of 4.6 years, from 2012-2014 to 2017-2019; strength, 400-m walk, chair stand, correlations between changes in physical activity and functional outcomes	Medication: pyridostigmine use decreased from 81% to 79%; 36% changed dose; multiple immunosuppressive changes. Timing: assessed on prescribed dose; interval NR. Adherence, withdrawals, and adverse events: NA, observational study	Report linkage: exact follow-up of two earlier Vinge baseline cohorts; the source does not establish overlap with Rahbek (S27) or national surveys. Data issues: older participants were preferentially lost; treatment changes were common; change in physical activity was not consistently associated with change in mobility or strength. Funding: study funding NR. Conflicts of interest: Thomsen reported an Alexion speaker fee; other industry relationships disclosed
Report citation(s): Anggarini et al. [16] (S37). Design/context: outpatient parallel RCT identified by citation search, Surabaya, Indonesia	23 randomized 11/12; 18 analysed 9/9. Adults with MGFA I–IIa; formal stability duration was NR	Low-intensity cycle ergometer 30 min three times per week for 8 weeks plus breathing exercises and standard care; comparator: breathing exercises and standard care	8 weeks; 2MWT, estimated VO2max	Medication: pyridostigmine, corticosteroids, azathioprine continued; changes were NR. Timing: exercise 1.5-2 h after dosing; testing was intended during the period of peak medication effect. Adherence: completer attendance was NR. Withdrawals: 5/23. Adverse events: reported events included discomfort in the intervention group, sinusitis, dyspnoea, hospitalization, and a later death judged unrelated; the source later stated that no adverse events occurred	Report linkage: different ethics approval from S03, but probable partial or substantial overlap could not be excluded because the final intervention groups had identical age and sex summaries; site re-enrolment unresolved. Data issues: five exclusions, adverse event statements conflicted, and presentation of pooled changes was ambiguous. Funding: none reported. Conflicts of interest: none reported
Report citation(s): Deliana et al. [39], Muslihah et al. [40], Putri et al. [41], and Amalina et al. [17]. (S38) Design/context: multiple reports apparently describing the same parent parallel-group RCT, Surabaya, Indonesia	Report-specific flow: Muslihah = 23 randomized and 22 analysed; Putri and Deliana = 20 analysed; Amalina = 20 randomized and 17 analysed. MGFA I–IIb, observed classes IIa and IIb; stability was NR	Cycle ergometer 30 min three times per week for 8 weeks plus lifestyle advice, breathing exercises, and the standard programme; comparator: breathing exercises, lifestyle advice, and the standard programme	8 weeks; no later follow-up; MG-ADL, MGC, and depression, spirometry, and surface electromyography (sEMG)	Medication: baseline pyridostigmine in some reports; usual treatment continued; changes and timing of dosing relative to sessions were NR. Adherence: Muslihah intervention group, 23.8/24 sessions; in Amalina, 8 of 9 intervention participants completed the 8-week programme; other fidelity was NR. Withdrawals: report flows were irreconcilable. Adverse events: reports state no exacerbation or exercise-related cardiac, respiratory, or musculoskeletal event; denominator for the parent trial unavailable	Report linkage: four reports share site, ethics 0601, intervention, comparator, duration, and closely similar baseline data; treated as one apparent parent trial. Data issues: recruitment dates and randomized and analysed participant flows conflict; Deliana inadequately described surface electromyography electrode placement, normalization, and processing; denominator for adverse events for the parent trial unavailable. Funding: none reported. Conflicts of interest: none reported

Table 2 provides the 75 result-specific risk-of-bias assessments and their concise rationales. Overall RoB 2 and ROBINS-I judgments follow the rules of the respective tools. For JBI appraisals, item responses were not summed; the overall concern labels are review-specific summaries and are not official JBI scores or categories. In the 2026 JBI analytical cross-sectional tool, X1-X6 address internal validity, X7 statistical-conclusion validity, and X8 reporting; in the 2025 JBI cohort tool, C11 addresses statistical-conclusion validity.

**Table 2:** Result-specific risk-of-bias assessments: 75 assessments across 38 study units and 47 reports (34 RoB 2, four ROBINS-I, 17 JBI cross-sectional, five JBI cohort, three JBI case series, one JBI qualitative, and 11 JBI quasi-experimental). Appraisal tools: RoB 2, Cochrane Risk of Bias 2; ROBINS-I, Risk Of Bias In Non-randomized Studies of Interventions; JBI, Joanna Briggs Institute. Response codes: L, low risk; SC, some concerns; H, high risk; M, moderate; S, serious; C, critical; Y, yes; N, no; U, unclear; N/A, not applicable. Domain/item codes: RoB 2, D1-D5 and DS; ROBINS-I, C, S, I, D, Mi, M, and R; JBI, X1-X8 cross-sectional, C1-C11 cohort, K1-K10 case series, Q1-Q10 qualitative, and Q1-Q9 quasi-experimental. Abbreviations: 2MWT, two-minute walk test; 6MWD, six-minute walk distance; 6MWT, six-minute walk test; ADL, activities of daily living; BDI, Beck Depression Inventory; CI, confidence interval; CMAP, compound muscle action potential; FEV1, forced expiratory volume in one second; FSS, Fatigue Severity Scale; FVC, forced vital capacity; HADS, Hospital Anxiety and Depression Scale; HR, hazard ratio; ITT, intention-to-treat; MDI, Major Depression Inventory; MEP/PEmax, maximal expiratory pressure; MFIS, Modified Fatigue Impact Scale; MG, myasthenia gravis; MG-ADL, Myasthenia Gravis Activities of Daily Living; MGC, Myasthenia Gravis Composite; MGFA, Myasthenia Gravis Foundation of America; MGQOL15/MG-QoL15, 15-item Myasthenia Gravis Quality of Life scale; MGQOL15F-r, revised French MGQOL15; MIP/PImax, maximal inspiratory pressure; MVV, maximum voluntary ventilation; Neuro-QoL, Quality of Life in Neurological Disorders; PAL, physical activity level; PBWSTT, partial body weight-supported treadmill training; PedsQL, Pediatric Quality of Life Inventory; PEF, peak expiratory flow; PFT, pulmonary function testing; QMG, Quantitative Myasthenia Gravis score; QoL, quality of life; RAS, rhythmic auditory stimulation; RCT, randomized controlled trial; RMET, respiratory muscle endurance training; RMT, respiratory muscle training; SD, standard deviation; sEMG, surface electromyography; SF-36, 36-Item Short Form Health Survey; VAFS, Visual Analogue Fatigue Scale; VO2max, maximal oxygen uptake.

Report or result assessed	Appraisal tool and ratings by domain or item	Overall judgment	Basis for judgment
S01 - Norling et al. [61]: effect of assignment on ring-derived physical activity and sedentary-time results	RoB 2 D1=SC; DS=N/A; D2=L; D3=SC; D4=L; D5=SC	Some concerns	Randomization reporting and the analysis population remain unclear, with differential loss across arms. The same device-derived OURA-ring method was applied across groups, and participants were blinded to ring output, supporting a low risk for outcome measurement; retention and wear time conditioning are reflected under missing data, while proprietary processing and multiple eligible analysis choices support concern about reporting. The typeset full text states that all randomized participants were included, but the principal analysis describes 72 completers and multiple imputation was reported as a sensitivity analysis; this does not resolve the analysis-population concern. The primary clinical outcomes were null, and several reported FSS, sleep, and medication values were internally inconsistent; device results remain secondary.
S01 - Norling et al. [61]: effect of assignment to physical activity coaching, sleep coaching, or passive monitoring on MG-ADL and FSS at the end of the intervention and at follow-up	RoB 2 D1=SC; DS=N/A; D2=L; D3=SC; D4=H; D5=SC	High risk	Sequence generation and concealment were not reported. The principal published analysis used 72 observed completers after 81 randomizations; multiple imputation was presented as a sensitivity analysis rather than a clearly implemented primary ITT analysis. Loss was differential across arms. MG-ADL and FSS were self-reported by participants aware of assignment. The registered outcome schedule, multiple eligible analyses, and internal data inconsistencies create reporting concern. The prespecified omnibus tests were null; a nominal pairwise MG-ADL contrast was unfavourable to physical activity coaching.
S03 - Kartika et al. [42]: effect of assignment on maximal walking speed and cadence at eight weeks	RoB 2 D1=SC; DS=N/A; D2=L; D3=SC; D4=H; D5=SC	High risk	Simple randomization was stated without concealment safeguards. Three of 20 randomized participants were excluded from the completer analysis. Walking speed and cadence were effort-dependent outcomes in an unblinded behavioural trial, so participant effort and assessor encouragement could plausibly differ by assignment. No protocol or registration was available, and several eligible outcomes and analyses were reported. The final intervention arm may overlap partially or substantially with Anggarini et al. [16], but exact independence could not be confirmed.
S03 - Kartika et al. [42]: effect of assignment to cycling plus breathing education versus breathing education on FSS at eight weeks	RoB 2 D1=SC; DS=N/A; D2=L; D3=SC; D4=H; D5=SC	High risk	Simple randomization was stated without concealment safeguards. Three of 20 randomized participants were excluded from the completer analysis; the reported reasons do not establish that missingness depended on the true FSS result, but this cannot be ruled out. FSS was reported by unblinded participants. No protocol or registration was available, and the reported direction of the FSS result was internally inconsistent. The final intervention arm may overlap partially or substantially with Anggarini et al. [16]; exact independence was not confirmed.
S04 - Chang et al. [71]: effect of assignment to inspiratory muscle training (IMT) plus aerobic exercise after discharge versus standard care on the registered primary MIP change at six weeks	RoB 2 D1=SC; DS=N/A; D2=L; D3=H; D4=SC; D5=SC	High risk	A computer sequence and sealed envelopes were reported, but full envelope safeguards were not described. Six of 60 randomized participants were excluded without a primary ITT or sensitivity analysis. MIP was assessor-blinded but depended on maximal voluntary effort. The trial was registered almost six years after completion. MIP was identified as the primary outcome, and the between-group change was null (p=0.18); no evidence establishes result-based selection of this primary result.
S04 - Chang et al. [71]: effect of assignment on internally consistent secondary changes in MEP, 6MWD, FVC, FEV1, FEV1/FVC, and PEF at six weeks	RoB 2 D1=SC; DS=N/A; D2=L; D3=H; D4=SC; D5=H	High risk	The same allocation and 6/60 complete-case exclusions apply. Secondary respiratory and walking outcomes were assessor-blinded but effort-dependent. Registration was retrospective; the primary MIP result was null; several favourable secondary outcomes were emphasized, and multiple analyses were available. The reported Borg score, percent predicted FEV1, effect size, and minimal clinically important difference values were internally inconsistent and could not be verified; only internally consistent changes from baseline are represented here.
S09 - Misra et al. [28]: effect of assignment on 6MWD and other objective clinical results at three months	RoB 2 D1=SC; DS=N/A; D2=L; D3=SC; D4=SC; D5=H	High risk	Allocation concealment was not reported, and two health-related withdrawals after randomization were excluded. Performance outcomes were influenced by effort, although outcome personnel were described as separate or blinded. Retrospective registration, multiple outcomes and time points, alternative raw-change and responder analyses, internally inconsistent P-values, and medication dose P-values incompatible with the reported means, SDs, and sample sizes created a high risk of selective reporting. The linked subanalysis by Tripathi et al. [29] of the intervention group was appraised separately with the JBI quasi-experimental checklist.
S09 - Misra et al. [28]: effect of assignment to graduated walking versus prescribed rest on the three-month MGQOL-15 responder outcome	RoB 2 D1=SC; DS=N/A; D2=L; D3=SC; D4=H; D5=H	High risk	Computer randomization was reported, but concealment was not. Open-label allocation and permitted medication changes did not by themselves demonstrate a deviation relevant to the result. One participant per group was excluded after a health-related withdrawal. MGQOL15 was reported by participants aware of allocation. Although these issues do not establish selection driven by the result, registration followed the trial and the calculation of sample size concerned continuous MGQOL15 change. Among multiple time points and analyses, the article foregrounded a selected responder outcome defined as improvement of more than 50%.
S11 - Chen et al. [69]: effect of assignment on modified-Barthel ADL through postoperative day five	RoB 2 D1=SC; DS=N/A; D2=SC; D3=L; D4=H; D5=H	High risk	Randomization, adherence, medication changes, and assessor blinding were incompletely reported, although all 80 participants appeared in the reported analyses. ADL required judgment and was assessed without reported blinding. No prospective analysis plan was available. The reported day 1 and day 3 SDs were inconsistent, and the associated p-values were not reproducible from either set of values; the prespecified hospital stay result was missing.
S11 - Chen et al. [69]: effect of assignment to the multimodal perioperative rehabilitation bundle versus control care on day 7 PFT and 6MWD endpoints	RoB 2 D1=SC; DS=N/A; D2=SC; D3=L; D4=SC; D5=H	High risk	The report states only that 80 participants were randomly divided 40/40. The reported analyses include all 80, with no evidence of missing outcome data, although a Consolidated Standards of Reporting Trials (CONSORT) flow was absent. Adherence, medication changes, assessor blinding, and possible differential attention or cointervention were insufficiently documented. PFT and 6MWD were objective or performance-based, but assessors were not reported as blinded. The unregistered analysis limited to endpoints creates concern; omission of hospital stay was treated separately as missing evidence and did not determine D5 for the reported result. Hospital stay was stated but not reported, and the published PFT p-values cannot be reproduced from the reported means, SDs, and group sizes. The exact inferential result remains unreconciled.
S12 - Mohamed et al. [72]: effect of assignment to PBWSTT plus active physiotherapy versus active physiotherapy alone on spirometry at 12 weeks	RoB 2 D1=L; DS=N/A; D2=L; D3=L; D4=SC; D5=SC	Some concerns	Independent sequence generation, CleanWeb concealment, blinded assessors, complete follow-up, unchanged medication/activity, and high attendance were reported. Spirometry was assessor-blinded but depended on maximal effort. The registry and paper differed in age range, programme duration, blinding description, and outcomes; registered repetitive nerve stimulation (RNS) and MGQOL15 were not reported. Numerous outcomes and exceptionally small reported SDs create concern about selection and precision.
S12 - Mohamed et al. [72]: effect of assignment on CMAP outcomes at 12 weeks	RoB 2 D1=L; DS=N/A; D2=L; D3=L; D4=L; D5=SC	Some concerns	Independent sequence generation, concealment, blinded neurophysiological assessment, complete follow-up, unchanged medication/activity, and high attendance were reported. CMAP measurement was objective. Registration-publication discrepancies, omission of registered RNS, multiple outcomes, and unusually small reported dispersion values create some concern about selection and quantitative precision.
S12 - Mohamed et al. [72]: effect of assignment on strength, 6MWD, and postural-stability outcomes at 12 weeks	RoB 2 D1=L; DS=N/A; D2=L; D3=L; D4=SC; D5=SC	Some concerns	Trial conduct and completeness were strong. Strength, walking, and balance were assessor-blinded but remained partly effort-dependent. Differences between registered and published outcomes, multiplicity, and exceptionally small reported standard deviations warrant concern but did not support a high-risk judgment.
S12 - Mohamed et al. [72]: effect of assignment on PedsQL fatigue and PedsQL total at 12 weeks	RoB 2 D1=L; DS=N/A; D2=L; D3=L; D4=H; D5=H	High risk	Trial conduct and completeness were strong, but these patient-reported outcomes were completed with knowledge of allocation in a behavioural intervention and were plausibly influenced by expectations or treatment experience. Differences between registered and published outcomes and multiplicity create additional reporting concern. The registered MGQOL15 outcome was not reported and was replaced by child-reported PedsQL fatigue and total scores. Participants knew assignment, and the reported variances were unusually small.
S13 - Andersen et al. [66]: within-participant effect of 110%-cadence RAS (FAST10) versus no RAS on 6MWD during one visit	RoB 2 D1=SC; DS=SC; D2=L; D3=L; D4=SC; D5=H	High risk	Randomized test order was reported without full allocation safeguards. Only 30 minutes separated two maximal walking tests; the model included order and found no order effect, but this did not fully exclude fatigue, learning, or carryover. No paired data were missing. The 6MWT was effort-dependent, and participants knew whether rhythmic auditory stimulation was present. After interim inspection, cadence was changed from 100% to 110% and sample size was adapted, making the favourable FAST10 result highly vulnerable to selection driven by the result.
S17 - Ceren et al. [73]: crossover effect of spinal stabilization exercise versus the comparator programme on objective pulmonary, strength, and walking results after each six-week period	RoB 2 D1=SC; DS=H; D2=SC; D3=L; D4=SC; D5=H	High risk	Safeguards for envelope allocation were unclear. The four-week washout was not established as adequate, and period, sequence, and carryover effects were not modelled. The registry and publication differed regarding the concurrent home programme. All 10 participants completed the study, and objective outcomes were assessed by blinded personnel, although pulmonary, strength, and walking tests were influenced by effort. Post hoc stopping, nonpaired crossover analyses, multiplicity, and an internally inconsistent reported value supported a high risk of selective reporting.
S17 - Ceren et al. [73]: crossover effect on FSS, VAFS, MGQOL-15, and other participant-reported results	RoB 2 D1=SC; DS=H; D2=SC; D3=L; D4=H; D5=H	High risk	The same carryover, intervention-contrast, analysis, and selective reporting concerns apply. Participants knew which programme they were performing, and the self-reported outcomes were plausibly influenced by expectations or treatment experience.
S19 - Birnbaum et al. [43] RCT: effect of assignment to home rowing versus usual care on the prespecified primary MGQOL-15-F result at month six	RoB 2 D1=L; DS=N/A; D2=L; D3=L; D4=H; D5=L	High risk	Central concealed randomization and imputed intention-to-treat analysis were used, and no deviation relevant to the result was demonstrated. MGQOL-15-F was reported by participants aware of allocation. Its measure, month 6 time point, and primary analysis of covariance (ANCOVA) were prospectively specified, supporting low risk for selection of this reported primary result.
S19 - Birnbaum et al. [43] RCT: effect of assignment on secondary MG-ADL results at months six and nine	RoB 2 D1=L; DS=N/A; D2=L; D3=L; D4=H; D5=SC	High risk	Central concealed randomization and imputed intention-to-treat analysis were used, and no deviation relevant to the result was demonstrated. MG-ADL was reported by participants aware of allocation. Multiple eligible secondary time points and analyses without multiplicity control create some concern about selection of the reported result.
S19 - Birnbaum et al. [43] RCT: effect of assignment on 6MWD, strength, and pulmonary results at month six	RoB 2 D1=L; DS=N/A; D2=L; D3=L; D4=SC; D5=SC	Some concerns	Randomization and allocation concealment were strong. Most month 6 objective denominators were 23 for exercise versus 19 for control, and physiological outcomes were not imputed. Performance and neuromuscular outcomes were effort-sensitive, and some evaluations were performed by the intervention physiotherapist. Numerous secondary outcomes were tested without multiplicity correction. The reported knee extension result has a 95% CI excluding zero but p=0.06 and is internally inconsistent.
S19 - Birnbaum et al. [43] RCT: effect of assignment on 6MWD, strength, and pulmonary results at month nine	RoB 2 D1=L; DS=N/A; D2=L; D3=SC; D4=SC; D5=SC	Some concerns	Randomization and concealment were strong, but month 9 non-imputed objective data were differentially incomplete: exercise generally retained 21-23 participants while control denominators fell to 16-18. Performance outcomes remained effort-sensitive, and numerous secondary outcomes/time points were tested without multiplicity correction. The month 6 6MWD signal was not sustained.
S27 - Rahbek et al. [62]: effect of assignment on MG-QoL15, MFIS, and MDI patient-reported results after 20 sessions	RoB 2 D1=SC; DS=N/A; D2=L; D3=H; D4=H; D5=SC	High risk	The same incompletely described allocation and three excluded randomized participants apply. These outcomes were self-reported by participants who knew their assigned training, making expectation- or experience-related measurement bias plausible. The exploratory trial was unregistered and reported multiple outcomes.
S27 - Rahbek et al. [62]: effect of assignment to progressive resistance versus aerobic training on assessor-blinded strength, performance, and QMG results after 20 sessions	RoB 2 D1=SC; DS=N/A; D2=L; D3=H; D4=SC; D5=SC	High risk	The random component and concealment within minimization were insufficiently described. Three of 15 randomized participants were excluded, including one participant with bulbar worsening and prednisolone initiation; only completers were analysed. Although the outcome assessor was blinded, performance and QMG remained partly effort-dependent. The exploratory trial was unregistered and reported multiple outcomes. Feasibility, adherence, and adverse events were assessed separately in the safety extraction.
S32 - Fregonezi et al. [67]: effect of assignment on PImax and PEmax at eight weeks	RoB 2 D1=SC; DS=N/A; D2=L; D3=SC; D4=SC; D5=SC	Some concerns	Sequence generation and concealment were not reported. Whether two pre-programmed withdrawals occurred before or after randomization was unclear. Technicians were blinded, but PImax and PEmax depended on maximal voluntary effort, and compliance was not quantified. No protocol or registry was available. Baseline MG-severity counts were arithmetically inconsistent between arms.
S32 - Fregonezi et al. [67]: effect of assignment on respiratory endurance, MVV, spirometry, and lung volume outcomes at eight weeks	RoB 2 D1=SC; DS=N/A; D2=L; D3=SC; D4=SC; D5=SC	Some concerns	The same allocation, withdrawal-timing, effort-dependence, adherence, and prespecification concerns apply. The full text reports no significant between-group respiratory endurance effect, MVV improvement only within the training group, and no meaningful spirometric/lung volume benefit. These null controlled outcomes must not inherit the favourable PImax/PEmax interpretation.
S32 - Fregonezi et al. [67]: effect of assignment on the SF-36 role-physical domain at eight weeks	RoB 2 D1=SC; DS=N/A; D2=L; D3=SC; D4=H; D5=H	High risk	Allocation and withdrawal timing were incompletely reported. SF-36 was self-reported in an unblinded behavioural trial. Nine domains were available, but only the favourable role-physical domain was highlighted without a prospective analysis plan or multiplicity control, creating high risk of measurement and selective reporting bias.
S35 - Lohi et al. [65]: adapted assessment of the effect of assigning one arm and the contralateral leg to training on maximal isometric strength after 10 weeks	RoB 2 D1=SC; DS=N/A; D2=L; D3=L; D4=SC; D5=SC	Some concerns	This is an explicitly adapted, unvalidated RoB 2 application because no dedicated randomized body-part variant exists. The side-randomization method and concealment were incompletely reported, but all 11 participants contributed results. Failure to deliver the planned elbow-extension dose and possible cross-education primarily affect interpretation of the assigned-side contrast and did not determine D2. Voluntary maximal effort, baseline side differences, paired-analysis requirements, and absent protocol information remain concerns.
S35 - Lohi et al. [65]: adapted assessment of the effect of body side assignment on fatigability after 10 weeks	RoB 2 D1=SC; DS=N/A; D2=L; D3=L; D4=H; D5=SC	High risk	The same unvalidated body side design and reporting limitations apply. Fatigability relied strongly on voluntary effort and was incompletely measured and reported, making knowledge of training side likely to influence the result.
S37 - Anggarini et al. [16]: effect of assignment to low-intensity cycling versus control care on 2MWT and derived VO2max changes at eight weeks	RoB 2 D1=SC; DS=N/A; D2=L; D3=H; D4=H; D5=SC	High risk	Lottery allocation was reported without safeguards for concealment, although no relevant deviation from assignment was demonstrated. Five of 23 randomized participants were excluded, with imbalance and reasons related to health or noncompliance that could depend on outcome risk. The two-minute walk test was influenced by effort, VO2max was derived rather than directly measured, and assessment was unblinded. No protocol or registration was available, and the pooled-delta presentation was ambiguous. The final intervention group may overlap with Kartika et al. [42], but independence was not confirmed.
S38 - Amalina et al. [17]: effect of assignment on FVC, FEV1, and FEV1/FVC at eight weeks	RoB 2 D1=SC; DS=N/A; D2=L; D3=SC; D4=H; D5=H	High risk	Amalina et al. [17] analysed 17 of 20 participants, with one withdrawal from the intervention group and two participants in the control group missing testing after the intervention. Their work, travel, and personal reasons reduce but do not eliminate concern about missing data. Spirometry was influenced by effort and obtained by unblinded researchers. Favourable endpoints measured after treatment were emphasized despite baseline imbalance and the report's statement that changes did not differ significantly between groups, making selective reporting likely.
S38 - Deliana et al. [39]: effect of assignment on SEMG activation and endurance at eight weeks	RoB 2 D1=SC; DS=N/A; D2=L; D3=SC; D4=H; D5=SC	High risk	Deliana et al. [39] reported all 20 participants as analysed, but links to reports with different parent denominators leave uncertainty about the analysed subset. Surface electromyography depended on voluntary effort, investigators were unblinded, and electrode placement, normalization, processing, and derivation of activation and endurance values were insufficiently described. No protocol was available; many muscles, sides, constructs, and analysis choices were eligible, although selection based on the result was not established.
S38 - Muslihah et al. [40]: effect of assignment to cycling plus shared breathing/lifestyle care versus shared care on MG-ADL at eight weeks	RoB 2 D1=SC; DS=N/A; D2=L; D3=L; D4=H; D5=SC	High risk	Sequence generation and concealment were not described. No deviation relevant to the result was identified. Muslihah et al. [40] reported 22/23 analysed, with one control withdrawal for personal reasons unlikely to depend on the true MG-ADL result. MG-ADL was assessed in an unblinded behavioural trial. The absence of a protocol and the availability of endpoint, within-group, and change analyses create D5 concern.
S38 - Putri et al. [41]: effect of assignment on BDI at eight weeks	RoB 2 D1=SC; DS=N/A; D2=L; D3=SC; D4=H; D5=SC	High risk	The report states 20/20 were analysed, but its denominator conflicts with the linked parent trial reports, leaving uncertainty about inclusion in this result. BDI was reported by participants aware of allocation. No prospective analysis plan was available, and several eligible analysis approaches were possible.
S38 - Putri et al. [41]: effect of assignment on MGC at eight weeks	RoB 2 D1=SC; DS=N/A; D2=L; D3=SC; D4=H; D5=SC	High risk	Parent-trial denominators were irreconcilable across reports. MGC contains examination and symptom components and was assessed by unblinded investigators, making differential judgment plausible. The unregistered analysis limited to endpoints and the availability of alternative change analyses create D5 concern.
S16 - Otaka et al. [23]. Target association: higher versus lower accumulated billed inpatient rehabilitation intensity after ventilator weaning. Estimand: association with time to ADL score 0 within four weeks; no baseline assignment policy effect was identified.	ROBINS-I C=C; S=S; I=C; D=L; Mi=M; M=M; R=M	Critical risk	Exposure was accumulated and dichotomized after ventilator weaning, so recovery trajectory could determine subsequent rehabilitation dose while also defining the outcome. The higher-intensity group was older and more severely impaired, with higher rates of tracheotomy and steroid pulse therapy, longer pre-index ventilation, lower BMI, lower hospital volume, and longer hospitalization. Content, participation, and tailoring based on fatigability were unavailable. Baseline ADL and outcomes were substantially missing and imputed. Conventional HR = 0.69 (0.48-0.99) and weighted HR = 0.79 (0.56-1.12) differed. The administrative ADL scale was not MG-specific.
S21 - Hsu et al. [22]. Target comparison: initiation of prescribed home RMT plus usual treatment versus usual treatment among patients unwilling to train. Intended estimand: comparative 12-week change in maximal inspiratory pressure; the report did not validly estimate this effect.	ROBINS-I C=S; S=L; I=L; D=M; Mi=L; M=S; R=C	Critical risk	Treatment choice depended on willingness, motivation, baseline function, disease severity, and treatment; matching covered only age, sex, and BMI. Prescribed frequency was internally inconsistent, adherence and medication changes were unavailable, and effort-dependent outcomes were measured in unblinded participants. The paper reported separate within-arm pre-post p-values rather than a valid between-group change or interaction and emphasized favourable training-arm significance. The comparative causal effect is therefore not reliably estimable.
S23 - Freitag et al. [21] with the overlapping preliminary report by Rassler et al. [45]. Target comparison: RMET plus usual care versus no RMET. Available estimand: complete-case/as-observed 13-month comparison among completers, not an assignment policy effect.	ROBINS-I C=C; S=S; I=L; D=S; Mi=S; M=S; R=S	Critical risk	Preference allocation depended on motivation, time, willingness to undertake strenuous RMET, and previous favourable training experience, all of which also influence the effort-to-exhaustion endpoint. Nine of 10 participants in Rassler et al. [45] map to the final cohort; seven participants in the final cohort had previously participated in the 2007 programme reported by Rassler et al. [44], including one who later entered as a control. Six of 18 trainees and one of six controls were lost for prognosis-related reasons. Training, medication, and measurement timing were modified after illness; testing was influenced by effort, learning, and encouragement and selectively reported.
S26 - Ambrogi and Mineo [20]. Target comparison: 17 selected rehabilitation completers from 2005 to 2010 versus 17 propensity-matched historical controls from 1999 to 2004. Estimand: matched completer/as-treated contrast in postoperative length of stay.	ROBINS-I C=S; S=S; I=L; D=L; Mi=L; M=M; R=S	Serious risk	The historical comparator leaves serious era, perioperative care, discharge, and treatment confounding despite propensity matching. Two of 46 programme entrants unable to sustain rehabilitation were excluded, and only 17 of 44 completers entered the matched analysis. Length of stay was complete in the matched cohort, but discharge practice was unblinded and era-dependent. The selected matched subset, many available endpoints/time points, and absence of a prospective plan create serious reporting concerns. Published ICU/morbidity p-values and remission analysis were not reproducible and are not used as causal evidence.
S02 - Birnbaum et al. [60]: descriptive frequencies of study-specific barriers and facilitators	JBI Cross-sectional X1=Y; X2=N; X3=U; X4=U; X5=N/A; X6=N/A; X7=U; X8=Y	Moderate to high concern for descriptive measurement/reporting; no causal judgment	Eligibility and respondents were described, but anonymous self-selected recruitment had no population denominator; diagnosis and exercise status were self-reported, and the barrier instrument was specific to the study. Confounding items were not applicable to simple descriptive frequencies. Erroneous fatigue category data, reversed group proportions, and irreconcilable denominators reduced statistical and reporting confidence. This row describes limitations of frequency estimates, not an effect of exposure.
S02 - Birnbaum et al. [60]; associations of exercise status with MG-ADL, MGQOL15F-r, and Neuro-QoL fatigue	JBI Cross-sectional X1=Y; X2=N; X3=U; X4=Y; X5=U; X6=N; X7=U; X8=Y	High concern for associations	Validated patient-reported outcome measures were used, but exercise exposure and MG diagnosis were self-reported. Comparisons were essentially unadjusted despite systematic differences in disease stability and plausible confounding by severity, treatment, motivation, and function; cross-sectional direction cannot be established.
S06 - Alsop et al. [52]; physical activity and sedentary behaviour associations with fatigue, quality of life, walking limitation, and balance confidence	JBI Cross-sectional X1=Y; X2=N; X3=U; X4=Y; X5=Y; X6=N; X7=U; X8=N	High concern	Established instruments were used for physical activity, sedentary behaviour, and clinical outcomes, and the regression was adjusted for age, body mass index, and disease duration. MG diagnosis and stability were self-reported, and important residual confounders included current severity, treatment, mobility, and comorbidity. Several reported confidence intervals and p-values were mathematically incompatible. The linked qualitative follow-up was appraised separately.
S07 - Glaubitz et al. [59]: study-specific results for walking distance, perceived effect, and barriers	JBI Cross-sectional X1=Y; X2=Y; X3=N; X4=U; X5=N/A; X6=N/A; X7=N; X8=Y	Moderate to high concern for descriptive measurement/reporting; no causal judgment	The custom questionnaire lacked validation, and its distance variable was mislabelled as steps. Frequency categories, denominators, and duration counts were inconsistent. Confounding items are not applied to simple descriptive barrier frequencies, and this row is not interpreted causally.
S07 - Glaubitz et al. [59]; exercise dose associations with QMG, MG-ADL, and MG-QoL15	JBI Cross-sectional X1=Y; X2=Y; X3=N; X4=Y; X5=U; X6=N; X7=U; X8=N	High concern for associations	MG was clinically diagnosed, and clinical outcomes were measured with established instruments. Exercise exposure was measured with an unvalidated study questionnaire and a custom, nonstandard calculation of metabolic equivalents. Analyses were unadjusted and cross-sectional, registration occurred after recruitment began, multiplicity was not addressed, and several reported MG-ADL, exercise duration, confidence intervals, and p-values were internally inconsistent.
S14 - Andersen et al. [50] and Andersen and Vissing [51], national component	JBI Cross-sectional X1=Y; X2=U; X3=U; X4=Y; X5=Y; X6=Y; X7=U; X8=Y	Moderate concern	The large national survey used validated clinical outcomes and prespecified multivariable models. Modified SGPALS and International Physical Activity Questionnaire (IPAQ) remained self-reported; individual MG confirmation was incomplete, and complete-case selection and inconsistencies in ancillary data remained. Linked reports represent one parent survey.
S15 - Andersen and Vissing [51], clinic accelerometry; adjusted MG-ADL/activity associations	JBI Cross-sectional X1=Y; X2=U; X3=Y; X4=Y; X5=Y; X6=Y; X7=Y; X8=U	Moderate concern	MG was clinically verified; the seven-day ActiGraph protocol and MG-ADL measurement were rigorous, and prespecified models addressed the identified main confounders. Cross-sectional data do not establish direction or causality, and treatment and comorbidity may leave residual confounding. Recruitment overlapped in time with the national survey component, but participant overlap was not reported. The clinic sampling frame and refusal/exclusion flow were not reported. Several reported regression values were internally impossible, although the principal MG-ADL coefficients were coherent.
S19 - MGEX linked baseline report: Birnbaum et al. [30]; within-MG physical activity associations	JBI Cross-sectional X1=Y; X2=Y; X3=Y; X4=Y; X5=N; X6=N; X7=U; X8=Y	High concern for associations	Activity was measured with a validated accelerometer, but the small prerandomization subset excluded men after matching and had one unreconciled recording relative to the randomized report. The analysis was cross-sectional and did not adjust for MG severity, treatment, or comorbidity; controls came from an external database.
S19 - MGEX linked baseline report: Birnbaum et al. [30]; comparison of physical activity between participants with MG and manufacturer controls	JBI Cross-sectional X1=Y; X2=Y; X3=Y; X4=Y; X5=Y; X6=Y; X7=U; X8=Y	Moderate to high concern for the comparison	The prerandomization subset of 33 women with MG was compared with 66 external controls matched for age, height, and weight. Device measurement was objective, but exclusion of men, one unreconciled recording, and incomplete adjustment for disease severity, treatment, and comorbidity limited interpretation.
S20 - Ruiter et al. [57]; strenuous-activity and fatigue association	JBI Cross-sectional X1=Y; X2=U; X3=N; X4=Y; X5=Y; X6=U; X7=Y; X8=Y	Moderate to high concern for the association; causal direction not established	The registry analysis used a validated fatigue outcome and multivariable adjustment, but physical activity was measured with a crude, unvalidated self-report item and individual diagnostic verification was incomplete. Activity categories were coarse, treatment intensity and residual comorbidity confounding remained, and the cross-sectional design could not establish direction. The BMI standard deviation was also reported inconsistently.
S24 - Westerberg et al. [48]: comparisons of physical inactivity between participants with MG and population controls matched for age and sex	JBI Cross-sectional X1=Y; X2=Y; X3=U; X4=Y; X5=Y; X6=Y; X7=U; X8=Y	Moderate concern	The questionnaires were appropriate for group comparisons, but only responding population controls matched for age and sex were analysed, and MG and control response patterns could differ. The cross-sectional comparison was unadjusted for treatment, comorbidity, and socioeconomic factors, and physical inactivity was self-reported.
S24 - Sabre et al. [49]: comparison of physical activity between Sweden and Estonia	JBI Cross-sectional X1=Y; X2=Y; X3=U; X4=Y; X5=Y; X6=N; X7=U; X8=Y	High concern for the country comparison	The Swedish group is the same 40-person S24 cohort, compared with 36 Estonian patients. Participants were not matched, and physical activity differences were not adjusted for education, treatment, pyridostigmine dose, socioeconomic context, and other clinical differences. Activity was self-reported, and response/denominator limitations remained. This is a descriptive contextual country comparison, not an MG-specific causal effect.
S25/S28 linked baseline report: O'Connor et al. [31]: within-MG physical activity associations in pooled Uppsala/Jönköping baseline data	JBI Cross-sectional X1=U; X2=Y; X3=U; X4=Y; X5=N; X6=N; X7=U; X8=Y	High concern for associations	The 27-person sample exactly pooled all 14 Uppsala and 13 Jönköping entrants and is not a third cohort. Within-MG correlations were small and unadjusted, source eligibility and wear criteria were incompletely reproduced, and Abstract versus Results gave inconsistent MGC correlations, including a sign change for PAL. Confounding, multiplicity, and cross-sectional direction were not addressed.
S25/S28 linked baseline report: O'Connor et al. [31]: comparison of physical activity between participants with MG and a healthy reference group described as stratified or matched	JBI Cross-sectional X1=U; X2=Y; X3=U; X4=Y; X5=Y; X6=U; X7=U; X8=Y	High concern for the comparison	The same linked 27-person baseline sample was compared with a published healthy reference described as stratified/matched by age, sex, and weight. Recruitment, contemporaneity, wear protocol, and analytic comparability could not be fully verified, and the repeated reference structure was not modelled. The comparison is contextual rather than causal.
S25/S28 linked baseline report: O'Connor et al. [31]: MG versus published chronic obstructive pulmonary disease/mitochondrial-disease physical activity comparisons	JBI Cross-sectional X1=U; X2=Y; X3=U; X4=Y; X5=N; X6=N; X7=U; X8=Y	High concern for the comparisons	The linked 27-person MG baseline sample was compared with historical published disease cohorts without matching or adjustment. Differences in disease, sampling, age/sex, devices, algorithms, and wear criteria make the comparisons unreliable; the authors themselves cautioned against them. They provide descriptive context only.
S34 - Grohar-Murray et al. [58]: study-specific results for functional status and perceived helpfulness	JBI Cross-sectional X1=U; X2=N; X3=N; X4=U; X5=N; X6=N; X7=N; X8=N	High concern	Both the exposure and study-specific outcomes for function and perceived helpfulness were vulnerable to measurement error. Exercise users were compared with a denominator that included those users, totals across functional categories were incomplete, participant counts for exercise use could not be reconciled, and no strategy addressed confounding.
S34 - Grohar-Murray et al. [58]; exercise self-care association with validated fatigue	JBI Cross-sectional X1=U; X2=N; X3=N; X4=Y; X5=N; X6=N; X7=N; X8=N	High concern	The foundation postal survey used a convenience sample, self-reported diagnosis, a crude exercise item, overlapping comparison denominators, and no adjusted analysis. The fatigue instrument was established, but selection, confounding, and cross-sectional directionality remained major concerns. Counts of exercise users were irreconcilable, and exact reporting was incomplete.
S05 - Dimmick et al. [55]; daily associations of activity with fatigue and symptoms	JBI Cohort C1=N/A; C2=Y; C3=Y; C4=Y; C5=Y; C6=N/A; C7=U; C8=Y; C9=N; C10=N; C11=N	High concern	Twelve-week ActiGraph exposure and longitudinal modelling were strengths, and several prespecified covariates were considered. Only 16 of 30 entrants supplied sufficient data, the daily sliders relied on unpublished validation, and the analysis did not adequately address selective data availability. Same-day analyses could not establish direction; backward selection and extensive exploratory testing were used without multiplicity correction, and one inactivity standard deviation was arithmetically implausible.
S08 - Steyaert et al. [56]; association between daily step count and MG-ADL	JBI Cohort C1=N/A; C2=U; C3=N; C4=N; C5=N; C6=N/A; C7=Y; C8=Y; C9=N; C10=N; C11=N	High concern	MG-ADL itself is an established outcome, but optional step exposure was poorly specified; the analysis used a small selected subset, attrition was substantial, and repeated observations and confounding were inadequately handled. Step data were optional and available for only 26 participants, with 14 in the exacerbation comparison; heterogeneous device sources and no common wear protocol limit interpretation.
S08 - Steyaert et al. [56]; association between daily step count and self-reported exacerbation	JBI Cohort C1=N/A; C2=U; C3=N; C4=N; C5=N; C6=N/A; C7=N; C8=Y; C9=N; C10=N; C11=N	High concern	Optional step capture had no defined device, wear, or completeness protocol. Exacerbation was self-reported in the same check-in as symptom data, creating circular and unreliable outcome ascertainment. Attrition and optional-data selection were substantial, and repeated days were not analysed with an adequate clustered longitudinal model. Step data were optional and available for only 26 participants, with 14 in the exacerbation comparison; heterogeneous device sources and no common wear protocol limit interpretation.
S36 - Thomsen et al. [54]; comparison of longitudinal strength and mobility trajectories between participants with MG and controls	JBI Cohort C1=Y; C2=Y; C3=Y; C4=U; C5=U; C6=N/A; C7=Y; C8=Y; C9=Y; C10=U; C11=Y	Moderate concern	MG and controls came from the same population framework and were reassessed with comparable protocols over 4.6 years. Reasons for attrition and baseline differences were described, and group-by-visit mixed models with selected covariate or treatment analyses provided a partial confounding strategy. Treatment change, residual baseline differences, time-varying confounding, and informative loss were not fully controlled. The report follows earlier baseline cohorts and is not an independent new baseline sample.
S36 - Thomsen et al. [54]; correlations between changes in physical activity and changes in strength and mobility	JBI Cohort C1=N/A; C2=Y; C3=Y; C4=U; C5=N; C6=N/A; C7=Y; C8=Y; C9=Y; C10=U; C11=N	Moderate to high concern; high concern for causality	The Physical Activity Scale was applied at both visits and has general validation evidence, and reasons for attrition were explored. Analyses used unadjusted correlations among completers, with unclear denominators, no multiplicity correction, and no control for baseline severity, physical activity, treatment change, or comorbidity. Exposure and outcome changed over the same interval, and change in habitual activity was not consistently associated with change in mobility or strength.
S10 - Punga et al. [26]; descriptive feasibility, attendance, and tolerability	JBI Case Series K1=Y; K2=Y; K3=Y; K4=N; K5=N; K6=Y; K7=Y; K8=U; K9=Y; K10=U	Moderate to high concern for descriptive feasibility	Eligibility was defined, and all seven enrolled participants completed, but only seven of 13 invited participants volunteered, mainly from an earlier cohort; consecutive or complete inclusion was therefore not achieved. Exact overlap with the earlier cohort was not reported, attendance and some reported denominators were inconsistent, and fatigue and quality-of-life trajectories were mixed. Intervention efficacy was appraised separately with the JBI quasi-experimental checklist.
S29 - Polastri et al. [70]; two-case postoperative course	JBI Case Series K1=U; K2=Y; K3=Y; K4=N; K5=U; K6=Y; K7=Y; K8=U; K9=Y; K10=N	High concern	The evidence comprised two uncomplicated thymectomy cases without a defined consecutive sampling frame or evidence that all eligible patients were included. Natural postoperative recovery could not be separated from physiotherapy; follow-up ended during the admission; trajectories were aggregated; and one reported dyspnoea value exceeded the stated scale maximum.
S30 - Wong et al. [27]; descriptive feasibility, completion, and tolerability	JBI Case Series K1=Y; K2=Y; K3=Y; K4=U; K5=U; K6=Y; K7=Y; K8=U; K9=U; K10=U	Moderate to high concern for descriptive feasibility	Physician-confirmed stable MG and clinical information were adequately described, but consecutive and complete inclusion were not established. Six rather than seven participants completed the intervention, and one additional participant missed the later follow-up. Outcome reporting was incomplete. Feasibility findings remain descriptive, and intervention efficacy was appraised separately with the JBI quasi-experimental checklist.
S06 qualitative companion: Alsop et al. [53]	JBI Qualitative Q1=U; Q2=Y; Q3=Y; Q4=Y; Q5=Y; Q6=U; Q7=N; Q8=Y; Q9=Y; Q10=Y	Moderate concern	Semi-structured interviews and content analysis addressed barriers, enablers, and advice, and participant quotations supported the themes. No explicit philosophical stance was stated, researchers' cultural or theoretical positioning was limited, and the investigators did not adequately address their influence on data collection or interpretation. This linked report informs experience and advice only, not intervention efficacy. The qualitative sample was a selected 10-of-85 subset of the earlier quantitative project and was relatively active and independently mobile.
S09 RESTOREX linked report: Tripathi et al. [29]; pre-post MGQOL-15, HADS, MG-ADL, and walking results	JBI Quasi-experimental Q1=Y; Q2=N; Q3=Y; Q4=U; Q5=N; Q6=Y; Q7=U; Q8=U; Q9=U	High concern for causal clinical efficacy	Clinical outcomes were collected in the same selected uncontrolled subset. Self-reported and effort-dependent outcomes were unblinded, follow-up selection was insufficiently documented, and multiple within-group outcomes and biomarker correlations were explored without a prespecified controlled analysis.
S09 RESTOREX linked report: Tripathi et al. [29]; pre-post β-endorphin and δ-opioid-receptor results in the walking group subset	JBI Quasi-experimental Q1=Y; Q2=N; Q3=Y; Q4=U; Q5=N; Q6=Y; Q7=Y; Q8=U; Q9=U	High concern for a causal exercise effect	The report selected 15 participants from the parent walking group completers and did not retain the randomized rest-arm comparison; 12 healthy controls were informed of baseline biology only. Medication stability, subset selection, and a separate withdrawal flow were insufficiently reported. Laboratory measurement was suitable, but the one-group pre-post design and exploratory correlations cannot isolate an exercise effect. This is a linked report within S09, not a new study.
S10 - Punga et al. [26]; six-month objective performance/strength results	JBI Quasi-experimental Q1=Y; Q2=N; Q3=Y; Q4=N; Q5=N; Q6=Y; Q7=U; Q8=U; Q9=U	High concern for causal efficacy	All seven enrolled participants completed, and treatment was reported as unchanged, but there was no concurrent control or repeated baseline. The sample was highly selected; participants were recruited mainly from an earlier intervention study, but the exact number of returning participants was not reported. Assessment was unblinded, and several outcomes reflected progressively trained loads. Measures based on individualized loads, photographs, and videos lacked an independent standardized reliability framework. Multiple small-sample comparisons, internal numerical inconsistencies, and mixed patient-reported trajectories further limited interpretation.
S18 - Chang et al. [63]; pre-post QMG result at 24 weeks	JBI Quasi-experimental Q1=Y; Q2=N; Q3=Y; Q4=N; Q5=N; Q6=Y; Q7=Y; Q8=N; Q9=U	High concern for causal efficacy	There was no concurrent control or repeated baseline; treatment stability during follow-up was unverified, and QMG data were available for only 32 of 35 entrants without sufficient explanation. QMG was measured using an established protocol, but the unblinded uncontrolled comparison remained vulnerable to time effects, regression to the mean, and influence from participant effort. The post hoc comparison of higher and lower exercise exposure did not show a significant interaction for QMG or quality of life.
S22 - Farrugia et al. [64]; MFIS and FSS results at week 10	JBI Quasi-experimental Q1=Y; Q2=N; Q3=Y; Q4=N; Q5=N; Q6=Y; Q7=Y; Q8=N; Q9=U	High concern; feasibility only	The small uncontrolled multimodal programme used one baseline assessment and had one early withdrawal. MFIS and FSS are established instruments, but participants were unblinded, and there was no concurrent control. The physical component could not be separated from psychological, educational, breathing, relaxation, and group self-management components.
S22 - Farrugia et al. [64]; visual analogue fatigue result at week 10	JBI Quasi-experimental Q1=Y; Q2=N; Q3=Y; Q4=N; Q5=N; Q6=Y; Q7=U; Q8=N; Q9=U	High concern; feasibility only	The small multimodal programme had no concurrent control, one baseline assessment, and one early withdrawal. The VAFS direction was defined as 0 = worst and 10 = normal, but MG-specific reliability remained uncertain. The apparent improvement at week 10 was not maintained at follow-up; an unpaired test was used for repeated observations, and the physical component could not be separated from psychological, educational, breathing, relaxation, and group self-management components.
S25 - Westerberg et al. [47]; 30-second chair-stand result after 12 weeks	JBI Quasi-experimental Q1=Y; Q2=N; Q3=Y; Q4=N; Q5=N; Q6=Y; Q7=Y; Q8=N; Q9=U	High concern for causal efficacy	The volunteer cohort had no concurrent control or repeated pre-intervention series; 3 of 14 entrants were lost, and three cholinesterase-inhibitor dose reductions occurred. The chair-stand protocol and paired comparison were suitable, but time effects, regression to the mean, cointervention, and multiplicity prevent causal attribution.
S28 - Westerberg et al. [46]; 6MWD after 12 weeks	JBI Quasi-experimental Q1=Y; Q2=N; Q3=Y; Q4=N; Q5=N; Q6=Y; Q7=Y; Q8=N; Q9=U	High concern for causal efficacy	There was no control or repeated baseline; three of 13 entrants did not complete, and the timing of rituximab remained unclear. The six-minute walk test was measured consistently, but assessment was unblinded, and multiple exploratory outcomes, time effects, and possible treatment cointervention remained. The withdrawals involved vertebral fracture, spinal stenosis, and planned thymectomy, but event attribution and treatment details were incomplete.
S30 - Wong et al. [27]; balance, QMG, and 6MWT change after training	JBI Quasi-experimental Q1=Y; Q2=N; Q3=Y; Q4=N; Q5=U; Q6=Y; Q7=U; Q8=N; Q9=U	High concern for causal efficacy	There was no concurrent control. Five participants had two baseline assessments, but two had only one, so protection from a repeated baseline was incomplete. Six of seven completed the intervention, and one additional participant missed follow-up; outcome assessment was unblinded and susceptible to learning, while analyses relied on descriptive change and an author-defined 15% threshold. The report also used a misleading denominator for completers and contained two sign errors.
S31 - Rassler et al. [44]; post-training respiratory endurance time	JBI Quasi-experimental Q1=Y; Q2=N; Q3=Y; Q4=Y; Q5=N; Q6=Y; Q7=U; Q8=Y; Q9=U	High concern for efficacy; selected-subgroup feasibility only	All 10 participants entering active training completed with stable treatment, but only 10 of 20 selected individuals could or would train. There was no concurrent control or repeated pre-intervention series, and endurance time was highly sensitive to learning, motivation, and encouragement. Seven of the 10 entrants later participated in the linked long-term S23 programme, so participant samples are not independent.
S33 - Weiner et al. [68]; PImax after three months	JBI Quasi-experimental Q1=Y; Q2=N; Q3=Y; Q4=Y; Q5=N; Q6=Y; Q7=Y; Q8=U; Q9=U	High concern; exact severe group PImax result unusable	The same PImax procedure was used over time, but the severity-stratified study had no non-training control, unclear flow and adherence, no repeated baseline, and testing influenced by effort. A verified copy inconsistency between severe group PImax and FEV1 makes the reported severe group numerical result unusable and limits the validity of the statistical conclusions. Severe group PImax, severe group endurance, and moderate group dyspnoea values were internally inconsistent; claims about preventing crisis or ventilation were unsupported extrapolations.

Certainty of Evidence

Certainty was very low for every principal outcome and clinical context in Table 3. Evidence concerning habitual activity began at low certainty and was further limited by measurement and reporting problems, selection, attrition, dependence among observations, inconsistency, and uncertain direction. Intervention outcomes for function, respiration, and patient reports in stable adults were downgraded for very serious risk of bias, inconsistency, and imprecision. Evidence for pulmonary function and walking around thymectomy was downgraded to very low because the only RCT had a high risk of bias, unreconciled inferential data, a null six-minute walk result, and an unreported outcome for hospital stay.

**Table 3:** Certainty assessment for principal outcomes and clinical contexts using the GRADE framework. Abbreviations: 6MWD, six-minute walk distance; ADL, activities of daily living; AE, adverse event; DIG-MG, Digital Phenotyping and Lifestyle Intervention in Patients With Myasthenia Gravis; FEV1, forced expiratory volume in one second; FSS, Fatigue Severity Scale; GRADE, Grading of Recommendations Assessment, Development, and Evaluation; HRQoL, health-related quality of life; IMT, inspiratory muscle training; MG, myasthenia gravis; MG-ADL, Myasthenia Gravis Activities of Daily Living; MGEX, Benefits and Tolerance of Exercise in Myasthenia Gravis; MGQOL15, 15-item Myasthenia Gravis Quality of Life scale; MIP, maximal inspiratory pressure; NRSI, non-randomized studies of interventions; PFT, pulmonary function testing; PImax, maximal inspiratory pressure; RAS, rhythmic auditory stimulation; RCT, randomized controlled trial; RNS, repetitive nerve stimulation; SD, standard deviation.

Principal outcome and context	Evidence streams	Main reasons for downgrading	Certainty	Interpretation
Associations of habitual physical activity and sedentary behaviour with fatigue, MG severity, function, or quality of life	Observational stream only; 12 study components plus linked/overlapping baseline analyses	Low observational starting certainty; additional serious measurement and reporting limitations, selection/attrition, optional-device subsets, non-independence, and analysis problems; serious inconsistency between adjusted inverse associations and mostly null or heterogeneous within-person/longitudinal findings; imprecision and several internally inconsistent estimates	Very low	More active people with MG often reported better outcomes, but evidence does not show that increasing habitual activity causes improvement in fatigue, MG severity, function, or quality of life
Objective functional or exercise capacity outcomes after structured exercise in selected, clinically stable community-dwelling adults	Randomized and non-randomized streams assessed separately; uncontrolled and acute-cueing studies used only as context	Very serious risk of bias; serious inconsistency and imprecision; primary or larger controlled findings were frequently null or not sustained; small favourable results included effort-sensitive testing, completer analyses, invalid crossover analyses, and an acute RAS effect below external clinically important thresholds	Very low	It is very uncertain whether structured programmes produce clinically important functional benefit in selected, clinically stable community-dwelling adults
Respiratory muscle performance and pulmonary function outcomes in selected, clinically stable community-dwelling adults	RCT stream: MGEX [43], Fregonezi et al. [67], Ceren et al. [73], and the Surabaya pulmonary report [17]; non-randomized and uncontrolled respiratory studies as context	Very serious risk of bias, serious inconsistency, and serious imprecision; Fregonezi et al. [67] showed a small strength signal, but no controlled endurance or spirometric benefit; Hsu et al. [22] did not validly estimate a comparative change; the Leipzig reports [21,44,45] overlapped and were at critical risk; Weiner et al. [68] contained multiple unusable values	Very low	Respiratory strength or endurance signals exist, but clinically important benefit and generalizability remain very uncertain
Fatigue, HRQoL, MG-ADL, or perceived daily function after structured exercise in selected, clinically stable community-dwelling adults	Randomized and non-randomized streams assessed separately; patient-reported constructs interpreted separately	Very serious risk of bias from unblinded self-report, missing data, retrospective or absent registration, and selective analyses; serious inconsistency and imprecision. The primary patient-centred outcomes in DIG-MG [61] and MGEX [43] were null, while RESTOREX [28,29] and the small uncontrolled studies were at high risk of bias	Very low	It is very uncertain whether exercise improves fatigue, MG-ADL, MG-specific quality of life, or perceived daily function in selected, clinically stable community-dwelling adults
Pulmonary function and walking capacity around thymectomy	Chen et al. [69] perioperative RCT; Ambrogi and Mineo [20] comparison of matched completers with historical controls; Polastri et al. [70] two-case series	Very serious risk of bias and serious imprecision. Chen et al. [69] pulmonary function inference could not be reproduced from reported means, SDs, and sample sizes; hospital stay was omitted, and 6MWD was null. Ambrogi and Mineo [20] used historically selected completers and reported categorical p-values that were not reproducible; case evidence was noncomparative	Very low	A multimodal perioperative bundle may affect selected postoperative PFT values, but the exact effect, clinical importance, and exercise-specific contribution are very uncertain
Early recovery in activities of daily living around thymectomy	Chen et al. [69] perioperative RCT; Polastri et al. [70] two-case context	Very serious risk of bias: judgment-dependent unblinded ADL measurement, incomplete trial methods, conflicting SDs, unreproducible early p-values, omitted hospital stay evidence, and no prospective analysis plan; serious imprecision	Very low	It is very uncertain whether the perioperative bundle improves early postoperative ADL
Respiratory or functional recovery after hospital discharge for MG exacerbation	One post-discharge RCT by Chang et al. [71]; 60 randomized and 54 analysed	Very serious risk of bias and serious imprecision: six randomized participants excluded, registration almost six years after the trial, null registered primary MIP result, selected positive secondary results, internally inconsistent Borg and predicted FEV1 values, unverifiable effect size estimates and purported minimal clinically important differences, and no reliable interval estimates	Very low	IMT plus aerobic exercise may improve selected secondary outcomes after discharge, but the evidence is very uncertain, and the primary comparison of inspiratory strength showed no between-group difference
Activities-of-daily-living recovery during inpatient rehabilitation after myasthenic crisis	One administrative cohort by Otaka et al. [23]; low starting certainty	Critical confounding and intervention-classification problems because rehabilitation dose accumulated after weaning and could depend on recovery; substantial baseline imbalance, missing data, non-MG-specific outcome, unavailable programme content, and conventional versus weighted estimates with different precision	Very low	The association cannot establish whether higher rehabilitation intensity delays or improves recovery after myasthenic crisis
Functional, pulmonary, and patient-reported outcomes after juvenile MG rehabilitation	One active-comparator RCT by Mohamed et al. [72] (n=30)	Very serious combined risk of bias and serious imprecision: registered RNS and MGQOL15 outcomes omitted or substituted, effort-dependent performance measures and unblinded patient-reported outcomes, many analyses, no confidence intervals, and exceptionally small reported SDs whose meaning could not be verified	Very low	Added treadmill training may improve selected 12-week outcomes in stable adolescents, but a single small trial with an active comparator cannot support a broad conclusion about efficacy in juvenile MG
Serious adverse events, symptom worsening, withdrawal, and treatment modification during structured exercise in selected stable adults	Randomized and non-randomized safety streams were assessed separately; report-specific denominators and linked samples were retained	Incomplete and inconsistent surveillance; absence of systematic denominators; adverse events and treatment changes not prespecified; limited evidence in unstable or severe MG	Very low	Selected stable participants often completed programmes, but the evidence cannot exclude clinically important harms or establish safety in people with unstable or severe MG, bulbar or respiratory compromise, or increased risk of myasthenic crisis

Evidence after hospital discharge was rated very low because the registered primary maximal inspiratory pressure (MIP) result was null, attrition was substantial, secondary reporting was selective, and several reported outcome values were internally inconsistent. Evidence after myasthenic crisis remained very low because of critical confounding and concerns about reverse causality. The body of evidence for juvenile MG was rated very low because one small active comparator trial substituted registered outcomes and reported unverifiable dispersion. Certainty about harms was also very low because surveillance was incomplete and estimates of uncommon events were imprecise.

Discussion

Principal Findings

Across selected samples, substantial proportions of participants were inactive, did not meet activity recommendations, or reported barriers to exercise, although activity distributions varied by instrument and cohort [30,31,50-53,55,56,59,60]. Greater self-reported activity was associated with less fatigue or lower MG burden in several cross-sectional datasets, but reverse causality and residual confounding remain plausible; evidence from repeated measurements was sparse and heterogeneous [50,52,55,57]. Some small intervention studies reported favourable between-group findings for selected outcomes or endpoints, as well as within-group improvements, including a binary primary outcome among participants analysed according to protocol in RESTOREX. In contrast, the primary patient-reported outcomes in DIG-MG and MGEX were null [28,43,61].

The most defensible interpretation is that selected patients with stable, usually mild-to-moderate MG often completed individually tailored programmes. However, certainty regarding functional, respiratory, and safety outcomes was very low; the evidence does not establish a single effective prescription, population-wide safety, or a broad disease-modifying effect.

**Figure 2:**
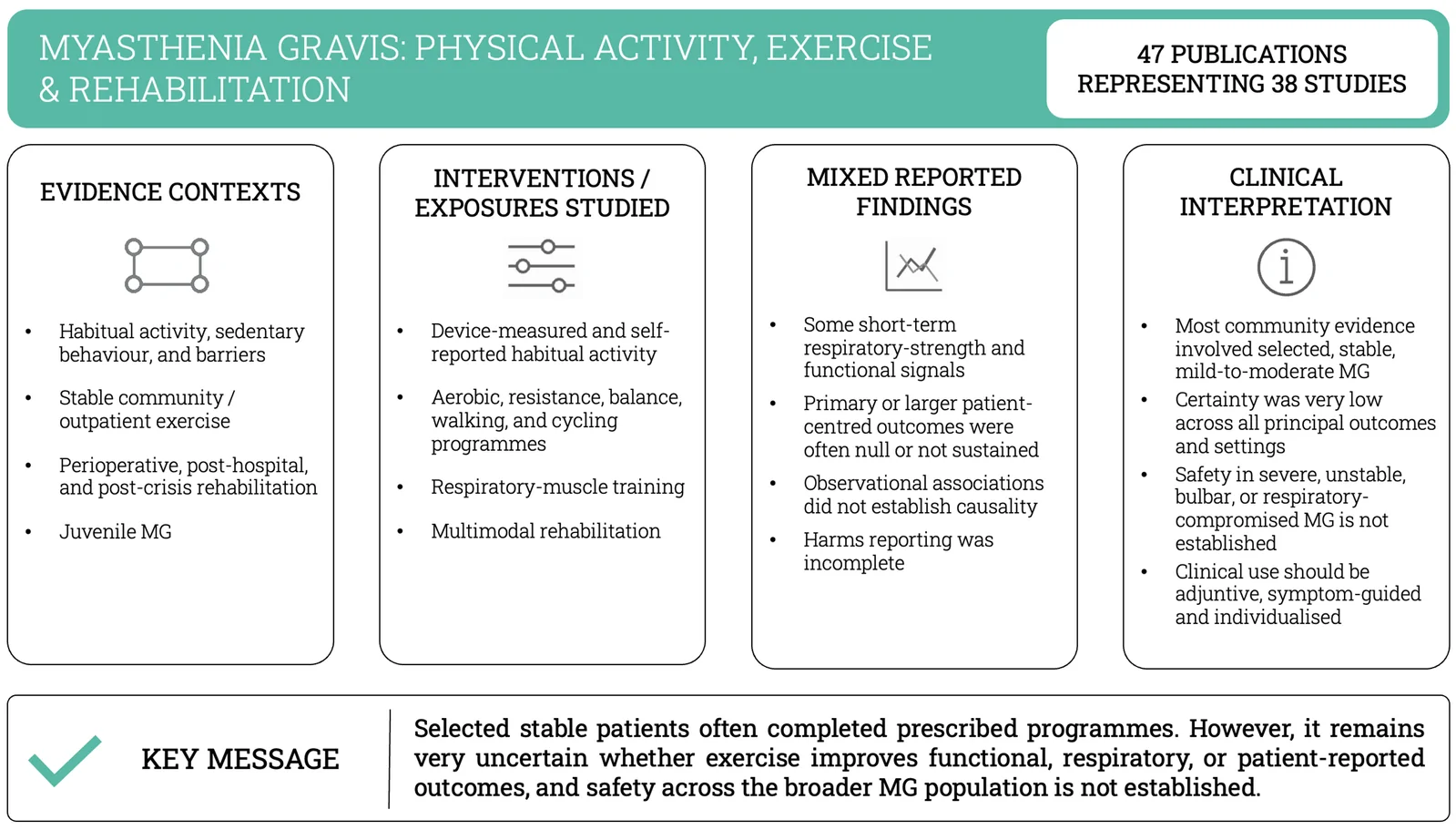
Conceptual summary of the reviewed evidence on physical activity, exercise, and rehabilitation in myasthenia gravis (MG). The figure distinguishes evidence contexts, exposures and interventions, mixed reported findings, and the cautious clinical interpretation supported by 47 reports representing 38 study units. The figure was created by the authors using Microsoft PowerPoint (Microsoft Corporation, Redmond, WA).

The clinical setting materially changed the interpretation of the evidence. Studies conducted around thymectomy evaluated multimodal programmes intended to preserve function during surgical recovery. The trial conducted after hospitalization evaluated inspiratory muscle training plus aerobic exercise following an exacerbation, whereas the administrative study of rehabilitation after myasthenic crisis measured billed intensity without identifying its therapeutic content [20,23,69-71]. Juvenile rehabilitation addressed a separate population and used an active comparator [72]. These evidence streams differed in population, comparator, additional care, outcomes, and recovery context. Combining them as equivalent evidence for chronic exercise in the community would therefore exaggerate consistency. Figure 2 summarizes the evidence contexts, exposures and interventions, mixed reported findings, and the cautious clinical interpretation supported by this heterogeneous evidence.

Comparison With Previous Reviews and Contribution of the Present Review

The 2021 focused review summarized 10 intervention studies involving 159 patients and recommended individually adapted training for mild-to-moderate MG [5]. The 2024 scoping review included 24 interventional and observational studies involving 456 patients across varied exercise modalities and emphasized heterogeneous protocols and limited adverse event reporting [6]. The 2025 rehabilitation meta-analysis included six controlled studies [7].

The 2026 physical activity and respiratory training review included 20 studies in its qualitative synthesis and 10 in its quantitative analyses [8]. A separate 2026 review restricted eligibility to eight controlled adult intervention studies and used descriptive synthesis because clinical and methodological heterogeneity precluded pooling [9]. Thus, eligibility criteria, included designs, and synthesis methods differed materially across prior reviews.

The present review complements rather than supersedes earlier reviews. It covers searches through July 2026 across three databases without a restriction on publication date. It integrates habitual activity, sedentary behaviour, barriers, and structured interventions. It also separates evidence from stable community populations, respiratory training, perioperative or acute recovery, and juvenile MG. Multiple publications are mapped to linked trials or cohorts, and unresolved overlap is identified.

The review also examines numerical inconsistencies; extracts disease stability, treatment, pyridostigmine timing, adherence, and adverse events; and applies risk of bias tools appropriate to each design together with certainty assessments by outcome. These procedures do not increase the certainty of the underlying evidence, but they reduce the risk of counting participants more than once or combining clinically distinct settings.

Interpretation of Efficacy and Observational Associations

For stable community-dwelling adults, activity and training should be framed as adjuncts rather than substitutes for MG-directed treatment. The null primary patient-centred outcomes in DIG-MG and MGEX show that feasibility and adherence do not guarantee clinical benefit [43,61]. RESTOREX's favourable binary responder result was at high risk of bias.

Registration followed the trial; the published responder estimand was not clearly prespecified before analysis, two randomized participants were excluded, treatment changes were permitted, and responder counts in the linked report could not be reconciled [13,28,29]. Positive secondary outcomes, uncontrolled improvements, and small-sample within-group changes should therefore be treated as hypothesis-generating.

Observational studies reported associations between activity and fatigue, disease burden, or quality of life, but they do not show that promoting activity causes these outcomes to improve [50,52,55,57]. Qualitative and survey studies separately described barriers and facilitators, including symptoms, fear of adverse consequences, exercise knowledge, practical access, social support, and specialist supervision [53,60].

Disease severity, depression, obesity, sleep, comorbidity, treatment, socioeconomic circumstances, and mobility can influence both activity and outcomes; higher activity may therefore partly reflect better underlying health rather than cause it. Longitudinal designs with repeated measurements should distinguish differences between participants from changes within participants. Objective monitoring should use transparent criteria for wear time and data completeness, and analyses should prespecify the time lag between activity and outcomes to strengthen causal inference.

Safety, Patient Selection, and Clinical Implications

Most community and outpatient intervention studies enrolled selected participants described as stable or well controlled and usually had mild-to-moderate generalized MG (Table 1). Stability was often incompletely operationalized, and severe or unstable disease, major bulbar or respiratory involvement, and substantial cardiopulmonary comorbidity were underrepresented. The separate trial conducted after hospital discharge was at high risk of bias [71], and the administrative association following myasthenic crisis was at critical risk [23]. These findings should not be extrapolated to recent crisis, marked bulbar or respiratory compromise, or uncontrolled comorbidity.

Reported serious events were few and often judged unrelated, but prespecified surveillance definitions and complete event reporting based on all randomized participants were usually absent. One feasibility trial reported possible training-related bulbar deterioration; the participant required prednisolone and withdrew [62]. Incomplete and heterogeneous surveillance prevents estimation of uncommon harms or safety across the wider MG population.

The evidence does not identify a universally effective modality, intensity, frequency, duration, progression rule, or stopping threshold. If activity or exercise is considered, symptom-guided individualization and coordination with MG-directed care are prudent. These precautions derive from MG management principles and have not been validated as exercise prescriptions in trials [1,2].

Baseline function, current disease stability, bulbar and respiratory involvement, comorbidity, concurrent treatment and treatment changes, access, and patient preference may be relevant when tailoring activity or exercise. New or sustained weakness, dysphagia, or dyspnoea should prompt clinical reassessment. No trial randomized exercise timing relative to pyridostigmine, and reported pragmatic scheduling or exploratory observations do not establish an optimal interval.

Research Priorities

Future trials should use adequately powered, clinically characterized samples and, where sample size permits, prespecified stratification or subgroup analyses by MG phenotype and severity. Protocols should define frequency, intensity, time, type, progression, dose modification, and stopping rules; prespecify a primary estimand, clinically important thresholds, multiplicity handling, and follow-up after supervised support ends; and report concurrent medication, rescue treatment, and changes in disease state. Outcomes important to patients should distinguish MG-ADL from measures of disease status rated by clinicians, such as QMG or MGC, and should include fatigue, MG-specific quality of life, objective function, and respiratory outcomes when relevant.

Activity dose and adherence should be reported as process measures. Harms surveillance should use prespecified definitions and denominators based on all enrolled or randomized participants for symptom exacerbation, bulbar or respiratory deterioration, falls, crisis, hospitalization, treatment change or interruption, modification of the exercise dose, withdrawal, and serious and non-serious events. Registries and publications should identify linked reports and participant overlap explicitly.

Limitations

This review was not prospectively registered, and no prospectively dated protocol was available to constrain post hoc decisions. The eligibility criteria, search methods, report linkage, risk of bias assessment, and certainty framework should therefore be regarded as post hoc. The search covered PubMed, Scopus, and Web of Science Core Collection but did not cover Embase, the Cumulative Index to Nursing and Allied Health Literature (CINAHL), SPORTDiscus, the Physiotherapy Evidence Database (PEDro), or trial registries. Backward citation searching was performed, but no separate search of forward citations or grey literature was undertaken.

Restricting eligibility to full reports in English created a risk of language bias: one potentially relevant record in Chinese was excluded before retrieval, and two full reports in Russian were excluded after retrieval. One older report of ventilatory training in a mixed population with neuromuscular disease was not retrieved. The requirement that exercise-related information be a central study focus may also have omitted useful contextual evidence. The search strategy was not peer-reviewed by an information specialist, and study selection, extraction, risk of bias, and certainty judgments were not independently duplicated; these limitations increase the risks of missed studies, assessment error, and subjective judgments.

Clinical and methodological heterogeneity was substantial. Many samples were small and clinically selected. Participants were often described as stable, although stability definitions were inconsistent or absent. Evidence concerning habitual activity was predominantly cross-sectional, and several intervention reports lacked concurrent controls, robust allocation, appropriate paired or longitudinal analyses, or complete reporting based on all randomized participants.

Linkage of overlapping reports, including probable but unconfirmed overlap, required judgment and may affect assumptions about participant independence even though the count of 38 study units was retained. Numerous internal source inconsistencies could not be corrected without raw data or author clarification. Adverse events, adherence, treatment changes, and pyridostigmine timing were often incompletely reported. We therefore judged quantitative pooling inappropriate and relied on narrative synthesis. Certainty was very low across all assessed outcome and context domains.

## Conclusions

In several observational datasets, greater self-reported habitual activity was associated with less fatigue, lower disease burden, or better patient-reported function, but other studies found no association. In one 12-week device cohort, only 16 of 30 enrolled participants entered the analytic sample, and no participant had complete data for all 84 days. One association within participants linked more moderate-to-vigorous physical activity with less fatigue, whereas most relationships between activity and symptoms within participants were null or inconsistent. These predominantly cross-sectional or contemporaneous associations remain vulnerable to confounding, selection, and reverse causality and do not show that increasing activity improves outcomes. For structured exercise and respiratory muscle training in selected community or outpatient adults, certainty was very low: favourable results for selected outcomes coexisted with null controlled primary outcomes, high risk of bias, imprecision, and heterogeneous interventions. It remains very uncertain whether these programmes improve objective function, respiratory performance or pulmonary function, fatigue, MG-ADL, or MG-specific quality of life.

The evidence does not establish a universal exercise prescription, population-wide safety, or the absence of uncommon clinically important harms. Evidence concerning perioperative care, recovery after crisis or hospitalization, and juvenile populations addresses distinct groups and should be interpreted within each clinical setting. Selected patients often completed prescribed programmes, but completion in small, clinically selected cohorts is not proof of efficacy or safety. If exercise is used clinically, it should remain adjunctive to MG-directed care and individualized to current disease status and comorbidity. Future studies require adequately powered and representative samples, prespecified and reproducible interventions and outcomes, transparent reporting of links between publications, appropriate longitudinal or crossover analyses, longer follow-up, and systematic reporting of adherence, treatment changes, and adverse events using all randomized participants as the denominator.

## Appendices

Reports excluded after assessment and one unretrieved report

Reports excluded after assessment of the full report and the unretrieved report are listed in Table 4.

**Table 4:** Reports excluded after assessment of the full report and the unretrieved report, with the primary reason for exclusion or non-retrieval. Abbreviations: MG, myasthenia gravis; PA, physical activity.

Report	Title	Screening outcome	Primary reason
Ducci et al., 2026	Prevalence and predictors of low bone mineral density in myasthenia gravis	Excluded	No eligible PA/exercise/rehabilitation exposure or intervention
Mishra et al., 2025	Development and feasibility assessment of a multimodal digital health technology for remote monitoring of symptoms in myasthenia gravis	Excluded	Feasibility report on digital monitoring without an extractable analysis relating physical activity to outcomes or evaluating a training intervention
Richardson et al., 2025	Levels of exercise exposure among people living with neuromuscular disorders: lessons learned from real-world data	Excluded	Wrong population: congenital myasthenic syndrome, not autoimmune MG
Xia et al., 2025	Observational and genetic evidence highlight the association of modifiable risk factors with the incidence and severity of neuroimmunological disorders	Excluded	Wrong population/question: incident-disease/Mendelian-randomization analysis
Vlasov et al., 2025	Results of medical and social research and assessment of quality of life in myasthenia gravis [Результаты медико-социального исследования и оценки качества жизни при миастении гравис]	Excluded	No eligible PA/exercise/rehabilitation exposure or intervention
Rushkevich et al., 2025	Results of rehabilitation of myasthenia gravis patients with subclinical respiratory disturbances [Результаты реабилитации пациентов с миастенией гравис и субклиническими дыхательными нарушениями]	Excluded	Full report not in English
Ala et al., 2024/2025	Assessment of nonmotor symptoms in myasthenia gravis: fatigue and disease burden	Excluded	Fatigue-only study without eligible PA exposure or training intervention
van der Woude et al., 2024	Feasibility and reproducibility of Isokinetic dynamometry in children with neuromuscular diseases	Excluded	Test/measurement study without eligible MG-specific PA or training effect
Leale et al., 2024	Physical activity in patients with neuromuscular disease three years after COVID-19, a longitudinal survey: the after-effects of the quarantine and the benefits of a return to a healthier life-style	Excluded	Mixed neuromuscular population; MG-specific data not separately extractable
Galievskaya et al., 2024	Quality of life and rehabilitation of myasthenia gravis patients with respiratory disturbances	Excluded	Full report not in English
Camdessanché et al., 2024	SPOON: an observational, cross-sectional study of perceptions and expectations of adults with generalised myasthenia gravis in France	Excluded	Broad perceptions/expectations study without eligible habitual-PA exposure or defined physical intervention; exercise barriers/advice were not an explicit primary focus
Li et al., 2023	Genetically predicted effects of physical activity and sedentary behavior on myasthenia gravis: evidence from Mendelian randomization study	Excluded	Mendelian-randomization design; not outcomes among people living with MG
Voet et al., 2022	Surface electromyography thresholds as a measure for performance fatigability during incremental cycling in patients with neuromuscular disorders	Excluded	Mixed-diagnosis exercise-test validation; MG data not separately extractable
Ng et al., 2021	Functional impairments and rehabilitation outcomes of patients with immunotherapy-induced acute inflammatory demyelinating polyradiculoneuropathy, myasthenia gravis, and myositis	Excluded	No defined, replicable MG-specific physical rehabilitation intervention/effect
Cabrita et al., 2021	Inspiratory muscle training in neuromuscular patients: Assessing the benefits of a novel protocol	Excluded	Mixed neuromuscular cohort; one MG participant and no separable MG outcome data
Tovazhnyanska et al., 2021	Rehabilitation of the patients with myasthenia gravis as an integral part of the patient’s treatment algorithm in the postoperative period	Excluded	No defined cohort rehabilitation intervention with extractable effect
Di Stefano et al., 2021	Significant reduction of physical activity in patients with neuromuscular disease during COVID-19 pandemic: the long-term consequences of quarantine	Excluded	Mixed neuromuscular-junction group; autoimmune-MG data not separately extractable
Dong et al., 2020	Gender differences in quality of life among patients with myasthenia gravis in China	Excluded	Broad social/leisure scale, not a defined PA or sedentary exposure
Veenhuizen et al., 2019	Self-management program improves participation in patients with neuromuscular disease: A randomized controlled trial	Excluded	Mixed neuromuscular trial; MG-specific results not separately extractable
Gross et al., 1993	The effect of ventilatory muscle training on respiratory function and capacity in ambulatory and bed-ridden patients with neuromuscular disease	Not retrieved	The full report could not be obtained through searches of publisher records and library holdings or attempts to contact the authors. Eligibility and the availability of separate MG data could not be established; the report was not counted among the 47 included reports or 38 study units

Exact database search strategies

The complete search syntax for each database, record totals, and final search date are provided in Table 5.

**Table 5:** Complete database search strategies. The searches were completed on 30 July 2026. No date, language, human, document type, or study design filters were applied in the database interfaces.

Database	Exact search strategy	Records exported
PubMed	( "Myasthenia Gravis"[MeSH Terms] OR myastheni*[tiab] ) AND ( "Exercise"[MeSH Terms] OR "Exercise Therapy"[MeSH Terms] OR "Motor Activity"[MeSH Terms] OR "Sedentary Behavior"[MeSH Terms] OR "Physical Therapy Modalities"[MeSH Terms] OR "Rehabilitation"[MeSH Terms] OR "Breathing Exercises"[MeSH Terms] OR "Sports"[MeSH Terms] OR exercis*[tiab] OR "physical activit*"[tiab] OR "habitual activit*"[tiab] OR "physical inactiv*"[tiab] OR sedentar*[tiab] OR "active lifestyle*"[tiab] OR "physical train*"[tiab] OR "aerobic train*"[tiab] OR "aerobic condition*"[tiab] OR "endurance train*"[tiab] OR "resistance train*"[tiab] OR "strength train*"[tiab] OR "strengthening exercis*"[tiab] OR "respiratory muscle train*"[tiab] OR "inspiratory muscle train*"[tiab] OR "expiratory muscle train*"[tiab] OR "respiratory muscle exercis*"[tiab] OR "inspiratory muscle exercis*"[tiab] OR "breathing exercis*"[tiab] OR "pulmonary rehabilitat*"[tiab] OR physiotherap*[tiab] OR "physical therap*"[tiab] OR rehabilitat*[tiab] OR prehabilitat*[tiab] OR treadmill*[tiab] OR "cycle ergomet*"[tiab] OR cycling[tiab] OR bicycl*[tiab] OR "walking program*"[tiab] OR "walking train*"[tiab] OR "balance train*"[tiab] OR "balance exercis*"[tiab] OR "stretching exercis*"[tiab] OR yoga[tiab] OR "tai chi"[tiab] OR qigong[tiab] OR "sport* activit*"[tiab] OR "sport* participation"[tiab] OR acceleromet*[tiab] OR pedomet*[tiab] OR "activity monitor*"[tiab] )	768
Scopus	TITLE-ABS-KEY( myastheni* AND ( exercis* OR "physical activit*" OR "habitual activit*" OR "physical inactiv*" OR sedentar* OR "active lifestyle*" OR "physical train*" OR "aerobic train*" OR "aerobic condition*" OR "endurance train*" OR "resistance train*" OR "strength train*" OR "strengthening exercis*" OR "respiratory muscle train*" OR "inspiratory muscle train*" OR "expiratory muscle train*" OR "respiratory muscle exercis*" OR "inspiratory muscle exercis*" OR "breathing exercis*" OR "pulmonary rehabilitat*" OR physiotherap* OR "physical therap*" OR rehabilitat* OR prehabilitat* OR treadmill* OR "cycle ergomet*" OR cycling OR bicycl* OR "walking program*" OR "walking train*" OR "balance train*" OR "balance exercis*" OR "stretching exercis*" OR yoga OR "tai chi" OR qigong OR "sport* activit*" OR "sport* participation" OR acceleromet* OR pedomet* OR "activity monitor*" ) )	1,169
Web of Science Core Collection	TS=( myastheni* AND ( exercis* OR "physical activit*" OR "habitual activit*" OR "physical inactiv*" OR sedentar* OR "active lifestyle*" OR "physical train*" OR "aerobic train*" OR "aerobic condition*" OR "endurance train*" OR "resistance train*" OR "strength train*" OR "strengthening exercis*" OR "respiratory muscle train*" OR "inspiratory muscle train*" OR "expiratory muscle train*" OR "respiratory muscle exercis*" OR "inspiratory muscle exercis*" OR "breathing exercis*" OR "pulmonary rehabilitat*" OR physiotherap* OR "physical therap*" OR rehabilitat* OR prehabilitat* OR treadmill* OR "cycle ergomet*" OR cycling OR bicycl* OR "walking program*" OR "walking train*" OR "balance train*" OR "balance exercis*" OR "stretching exercis*" OR yoga OR "tai chi" OR qigong OR "sport* activit*" OR "sport* participation" OR acceleromet* OR pedomet* OR "activity monitor*" ) )	830
